# Protein Posttranslational Modifications in Immunity: Molecular Mechanisms and Therapeutic Targets

**DOI:** 10.1002/mco2.70951

**Published:** 2026-08-30

**Authors:** Yuxing Yao, Jinxiu Qian, Qiqiong Liu, Wanting Ye, Ying Xing, Xiuyun Bai, Rongjun Deng, Jue Yang, Cheng Lu, Cheng Xiao, Yuanyan Liu

**Affiliations:** ^1^ School of Materia Medica Beijing University of Chinese Medicine Beijing China; ^2^ Institute of Basic Research in Clinical Medicine China Academy of Chinese Medical Sciences Beijing China; ^3^ Institute of Clinical Medicine China‐Japan Friendship Hospital Beijing China

**Keywords:** adaptive immunity, immune‐related diseases, innate immunity, molecular mechanisms and therapeutic targets, posttranslational modifications (PTMs)

## Abstract

The immune response is a highly dynamic and precisely controlled process, and relies on an intricate network of protein interactions to maintain its homeostasis. Protein posttranslational modifications (PTMs), by covalent addition of chemical groups or peptide chains, or by proteolytic cleavage, directly alter the structure, activity, electrical charge, thermal stability, and function of immune‐related proteins. Here, we enumerate the main PTMs in the immune system and highlight their global regulatory roles across innate immunity, T‐cell‐mediated cellular immunity, and B‐cell‐mediated humoral immunity, including phosphorylation, ubiquitination, SUMOylation, acetylation, methylation, glycosylation, and other new types. We focus on how these modifications regulate antigen presentation and recognition, immune signal transduction, antibody production and class switching, immune contraction, immune memory establishment, as well as immune tolerance and homeostasis. Critically, dysregulation of host protein PTMs disrupts immune homeostasis, which in turn induces numerous immune‐related diseases, including cancer, autoimmune diseases, and infectious diseases. By integrating mechanistic insights with emerging therapeutic strategies, this review provides a comprehensive PTM network that elucidates the molecular basis of immune‐related diseases and highlights promising immunotherapeutic strategies targeting these modifications.

## Graphical abstract

Protein posttranslational modifications, including phosphorylation, ubiquitination, SUMOylation, acetylation, methylation, glycosylation, and other new types, dynamically regulate antigen presentation and recognition, immune signal transduction, antibody production and class switching, immune contraction, immune memory establishment, as well as immune tolerance and homeostasis maintenance. Establish a foundation for the development of novel targeted immunotherapies.

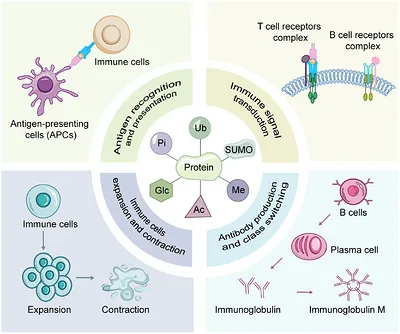

## 1 Introduction

The immune system is a sophisticated network in which organs, leukocytes, proteins, and soluble mediators coordinately orchestrate protective responses to fight pathogens and aberrant cells, while maintaining immune self‐tolerance to prevent autoimmunity. This intricate process demands rapid execution and robust self‐regulation [1]. However, transcriptional and translational changes alone are often insufficient to meet these dynamic demands. Intriguingly, protein posttranslational modifications (PTMs) significantly broaden the functional diversity of immune‐related proteins at the molecular level by covalent attachment of chemical groups and proteolytic cleavage, which are key regulatory factors in mediating immune response and influencing disease progression [2, 3].

Recent advances in proteomics, modification‐omics, and single‐cell technologies, the role of PTMs in immune regulation have progressively unveiled the roles of PTMs in immune regulation. Owing to their high dynamism and reversibility, PTMs govern receptor antigen recognition and presentation, immune signal transduction, and immune cell differentiation and memory throughout the immune response by modulating protein conformation, structure, subcellular localization, and intermolecular interactions [4, 5]. To date, numerous PTMs have been discovered, ranging from canonical types including phosphorylation, ubiquitination, SUMOylation, acetylation, methylation, and glycosylation to emerging modifications such as S‐nitrosylation, succinylation, lactylation, lysine malonylation, and citrullination [6]. They coordinate with each other and/or trigger intricate crosstalk‐cascades to regulate cellular phenotypes and biological processes, thereby providing organismal complexity. The interaction of PTMs operates at both microscopic and macroscopic levels. Microscopically, a particular PTM depends on other PTMs for its recruitment, initiation, and even activation. For instance, SUMOylation establishes noncovalent attachment with SUMO‐interacting motifs (SIMs), in the form of which expands the possibilities of protein–protein interactions, and enables crosstalk‐cascades between SUMOylation and other PTMs [7]. Macroscopically, given the dynamic and mutually responsive nature of immune processes, all PTMs ultimately act in a coordinated fashion to collectively participate in and influence the entire immune response, including innate immunity, T‐cell‐mediated cellular immunity, B‐cell‐mediated humoral immunity, and the crosstalk among them [8].

Although relevant research continues to accumulate, the distribution patterns and synergistic relationships among different PTMs across immune processes remain poorly defined, and an integrated regulatory framework spanning diverse disease contexts is still lacking. In particular, how PTM networks are coordinated within and between immune cell populations during complex homeostatic processes, such as immunological synapse formation and antigen presentation, and how this interconnection undergoes pathological remodeling in settings like tumor immune evasion, autoimmunity, and chronic infection, remains lacking a systematic summary. Consequently, existing knowledge has yet to coalesce into a unified mechanistic paradigm, which limits our comprehensive understanding of immune homeostasis disruption and impedes the development of mechanism‐guided immunotherapies.

Here, various PTMs go beyond merely supporting immune‐related protein synthesis and molecular mechanism regulation to maintain immune homeostasis; conversely, dysregulations in PTMs are also critical elements in the pathogenesis of various immune‐related diseases. Researchers have indicated that abnormal PTMs are involved in pathological processes, including cancer immune evasion [9], disrupted immune tolerance in autoimmune diseases [10], and evasion of host clearance by pathogenic microorganisms [11]. Consequently, PTMs have emerged as highly promising therapeutic targets that transcend the intrinsic limitations of genetic encoding. In recent years, small molecule inhibitors (SMIs), biological agents, and other technological interventions targeting PTMs have achieved great success [12, 13]. Based on these findings, this article reviews the major PTMs in immunity and the molecular mechanisms by which PTMs mediate immune responses, including antigen recognition and presentation, immune cells activation, differentiation and function, antibody production and class switching, immune contraction, and the establishment of immune memory. Meanwhile, this review further summarizes the connections between PTMs imbalance and immune‐related diseases, including cancer immune evasion, autoimmune abnormalities, and the persistence of infectious diseases, and outlines potential intervention strategies targeting PTMs enzymes and related modification pathways. By establishing an immune regulation framework centered on PTMs, this review is expected to provide a new theoretical basis and research perspective for immune homeostasis regulation and precision immunotherapy.

## 2 Major PTMs in Immunity

**FIGURE 1:**
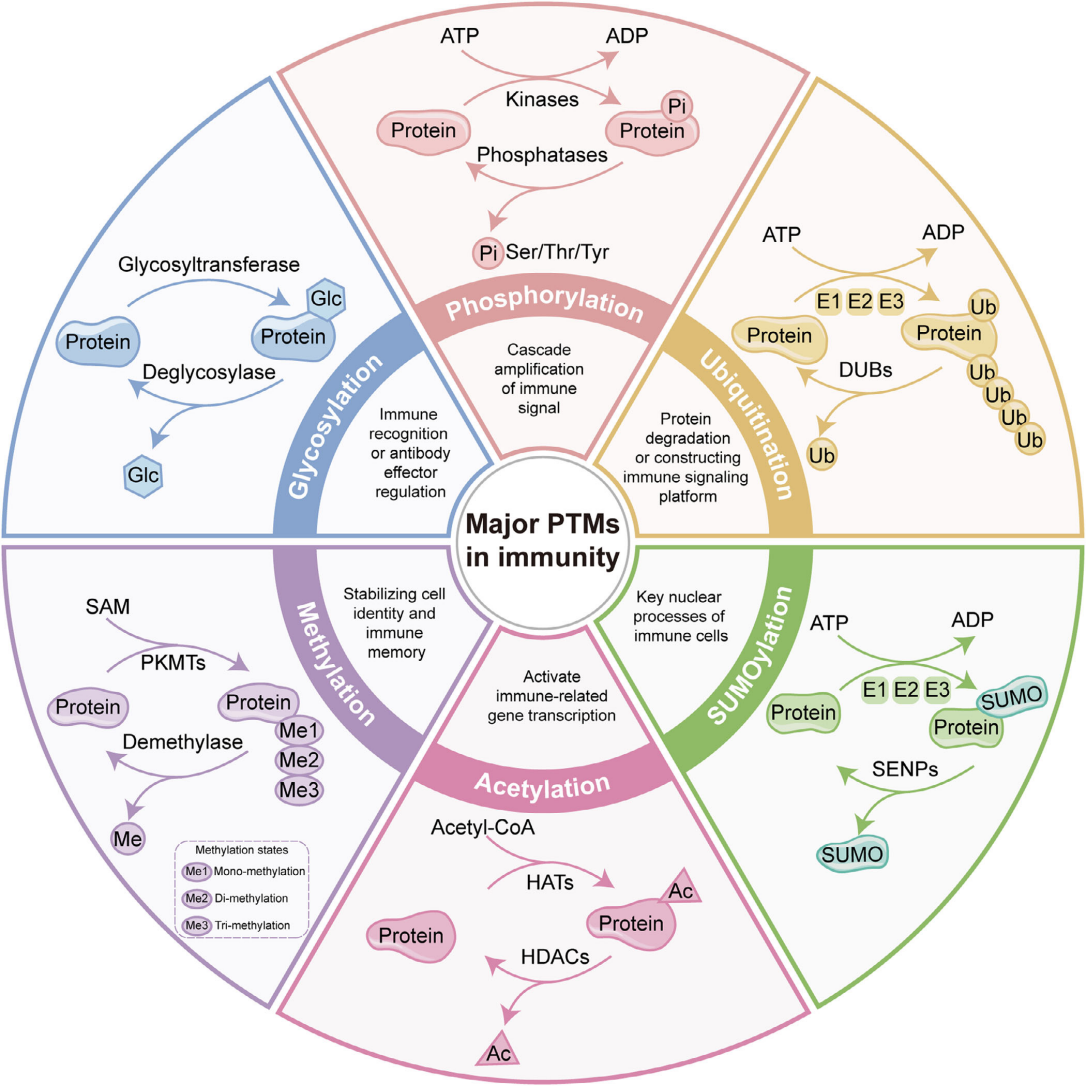
Common protein posttranslational modifications (PTMs) in immunity and their roles in immunity. Phosphorylation mediates kinase cascade reactions, regulating receptor complex assembly, transcription factor activation, and cytoskeleton remodeling. K48‐linked ubiquitin chains mediate the termination of immune signaling, while K63‐linked ubiquitin chains mediate the assembly of immune signaling complexes. SUMOylation usually inhibits transcription factor activity, regulates nucleo‐cytoplasmic shuttling, stabilizes protein‐protein interactions, and counteracts ubiquitin‐mediated degradation. Acetylation regulates histone epigenetics and directly modifies non‐histone proteins, affecting DNA binding and transcriptional activity. Methylation regulates the activity of immune‐related transcription factors and participates in cytokine receptor signaling. Glycosylation regulates immune receptor stability, membrane expression, ligand recognition, cell adhesion, and antibody effector functions. Ac, acetyl group; ADP, adenosine diphosphate; ATP, adenosine triphosphate; DUBs, deubiquitinating enzymes; E1, activating enzyme; E2, conjugating enzyme; E3, ligase enzyme; Glc, glycosyl group; HATs, lysine acetyltransferases; HDACs, lysine deacetylases; Me, methyl group; PKMTs, protein lysine methyltransferases; Pi, inorganic phosphate; Ub, ubiquitin; SENPs, SUMO‐specific proteases; SUMO, small ubiquitin‐like modifier.

PTMs significantly expand the intricacy of the human proteome and guarantee that each protein is expressed in its properly conformation at specific time points in particular cells of the body. Immune response, a precisely regulated cascade of protein reactions, mobilizes extensive PTMs to ensure accurate immune‐related protein expression and enable dedicated immune surveillance. Any addition of immune‐related chemical groups or proteins, as well as proteolytic cleavage, may modulate their protein stability, efficacy, localization, immunogenicity, and interactions with others [14]. Accumulating evidence from previous studies has demonstrated that several PTMs, including phosphorylation, ubiquitination, SUMOylation, acetylation, methylation, and glycosylation, can coordinately modulate the development, activation, and functional homeostasis of both innate and adaptive immune cells, thereby maintaining immune system homeostasis and mediating host defense against invading pathogens (Figure 1).

### 2.1 Phosphorylation

Protein phosphorylation is the most widespread and indispensable PTM in eukaryotes. Nearly 30% of proteins in mammalian organisms undergo phosphorylation, including those in all types of immune cells [15]. Phosphorylation, as a highly efficient energy‐dependent and extremely responsive PTM, can rapidly regulate the functions of cytoplasmic and nuclear target proteins of immune cells, in coordination with other PTMs, construct a precise immune signaling network [16]. It finely regulates immune cells by affecting the activity [17], stability [18], conformation of immune‐related proteins, and protein–protein interactions (PPIs), among other factors [19].

Protein phosphorylation denotes the process in which the terminal phosphate of ATP is covalently transferred to specific amino acid residues of a substrate protein mediated by protein kinase [20]. It target sites include Ser, Thr, Tyr, His, and Pro residues, but the phosphorylation of immune‐related proteins primarily found on Ser, Thr, and Tyr residues, and tyrosine phosphorylation is dynamically abundant in immune systems [21]. For example, tyrosine phosphorylation of STAT determines the lineage differentiation of CD4^+^ T cells [22]. In contrast, the reverse phosphorylation is referred to as dephosphorylation, which is a phosphatase‐mediated enzymatic reaction [23]. As a PTM that highly depends on ATP, it can be affected by changes in the ATP/ADP ratio, which influence the balance between kinases and phosphatases, while lactate can influence energy metabolism. The accumulation of lactate in the tumor microenvironment (TME) can affect the phosphorylation state of immune cells, fostering the establishment of immunosuppressive environment [24], such as lactic acid drives the conversion of macrophages to M2 by regulating the phosphorylation level of STAT3 signaling pathway [25].

In conclusion, the phosphorylation system is composed of four key elements: kinases that catalyze the covalent attachment of phosphate groups, phosphatases that mediate the removal of phosphate groups, substrate proteins that undergo specific phosphorylation, and effector binding proteins that specifically recognize phosphorylated signals and mediate downstream cellular processes [26]. This system gradually activates immune pathways by changing the conformation of immune‐related proteins, recruiting downstream signaling molecules to form immune signal complexes [27]. Ultimately, phosphorylated signals are transmitted to the immune cell nucleus, activating transcription factors (TF) that mediate cytokine and chemokine gene expression [3].

### 2.2 Ubiquitination

As a multi‐step and conserved PTM, ubiquitination not only influences the function of immune cells by modulating protein degradation [28] but also regulates key signaling molecules including kinases, adaptor proteins, and TFs to precisely control the intensity and timing of immune responses, and serves a pivotal function in mediating immune tolerance and maintaining homeostasis [29].

Ubiquitination is the process whereby ubiquitin (Ub) units (monomers or chains) are covalently linked to protein residues [30]. Ub is a 76‑amino‑acid small protein with a highly conserved sequence across eukaryotes and contains an N‐terminal methionine (Met1) and seven lysine residues K6, K11, K27, K29, K33, K48, and K63 through which the ubiquitin chain is lengthened. Among them, K48‐ and K63‐linked ubiquitination is frequent in immune processes [31], and Ub linked to each other via K48 represents the canonical signal for Ub‐proteasome system (UPS) [32], such as K48 ubiquitination of the STAT3 protein can terminate the continuous activation of the JAK/STAT signaling pathway [33]. While those linked via K63 not only amplify immune signals [34], for example, the K63‐linked Ub residues added to the lysine of RIG‐I enhance the induction of type‐I interferons (IFNs), but also clear damaged organelles by the lysosomal degradation pathways [35].

The ubiquitination process includes a series of reactions mediated by Ub activating enzyme (E1), Ub conjugating enzyme(E2), and Ub ligase enzyme (E3) [36]. E1 enzymes first activate Ub in an ATP‐hydrolyzing reaction by forming a high‐energy thioester bond, and then transferred to the active‑site cysteine of the E2 enzyme. Finally, E2 enzymes facilitate substrate ubiquitination assisted by E3 enzymes [37]. Ubiquitination in immune cells is substrate specific and is determined by E3 ligases. As a reversible PTM, deubiquitinating enzymes (DUBs) mediate reversible modification by removing conjugated Ub from target proteins [38]. Some ubiquitinated proteins alter their activity and localization, while most undergo degradation by UPS or lysosomal degradation pathways. Thus, they help prevent excessive or insufficient activation of immune response and maintain immune homeostasis [39].

Based on the linkage type, Ub chains can be divided into monoubiquitination (a lysine), multimonoubiquitination (multiple lysines), and polyubiquitination (forming Ub polymer). Monoubiquitination regulates the intracellular localization and transport of major histocompatibility complexes (MHC) molecules [40], whereas multimonoubiquitination usually enhances the initial transmission of immune signaling molecules [41]. In addition, Ub can also modify the Met1 of a target protein, a process known as linear ubiquitination and primarily participates in upregulation of immune signal transduction cascades, such as nuclear factor‐kappaB (NF‑κB) pathway [42].

### 2.3 SUMOylation

Small ubiquitin‐like modifier (SUMO) modification (also termed SUMOylation), a dynamic and reversible process of PTM, is pivotal for the flexible regulation of key nuclear processes of immune cells, such as membraneless organelle formation, genome regrogramming, and cell cycle progression, either by itself or through extensive PTM crosstalk [7].

The reversible modification process of SUMO is regulated by a cascade of enzymatic reactions akin to ubiquitination, including maturation, activation, and conjugation [43]. SUMO maturation involves the cleavage of the C‐terminus by SUMO‐specific proteases (SENPs), exposing the di‐glycine motif indispensable for SUMO conjugation [44]. Next, SUMO activation is initiated by an E1 activating enzyme. Then the E2 enzyme Ubc9 for SUMO conjugation receives SUMO from E1, catalyzing the covalent attachment of SUMO to the substrate [45]. E3 ligases facilitate the transfer of SUMO from Ubc9 to the lysine residues of target proteins. SENPs remove SUMO from target proteins by hydrolyzing the isopeptide linkage [46]. In certain tumor models, overexpression of SUMO proteins and abnormal SUMOylation of target protein may cause an increase in immunosuppressive cytokines, thereby helping tumors evade immune surveillance [47].

The SUMO protein family comprises five paralogs in mammals, SUMO1‐5 [48]. SUMO1 mainly ensures the normal function of immune cells, while SUMO2/3 contribute to host defense. SUMO4 is only expressed in specific organs, including kidneys, spleen, and lymph nodes [49], and SUMO conjugation can be monoSUMOylation (a lysine), multiSUMOylation (multiple lysines), or polySUMOylation (forming SUMO chains). Although SUMO and ubiquitin exhibit only 18% amino acid sequence identity, they both contain a typical ββαββαβ fold and a di‑glycine motif C‑terminal [50]. Unlike ubiquitinated proteins, which are usually degraded, SUMOylated proteins are more stable [51]. Taking the Lys21 site of inhibitor of kappaB alpha (IκBα) as an example, this site can undergo both SUMOylation and ubiquitination, but SUMOylation antagonizes the occurrence of ubiquitination [52].

SUMOylation can directly covalently attach SUMO to target proteins or indirectly regulate their biological functions by the SIMs [53]. The non‐covalent interactions mediated by SIM was proposed as characteristic property of SUMOylation, which establishes noncovalent attachment with SIMs and, in this form, expands the possibilities of PPIs and enables crosstalk between SUMOylation and other PTMs. The SUMO:SIM interactions facilitate the formation of regulatory complexes that provide a versatile PPI manner and enable crosstalk with other PTMs [54]. This interaction helps recruit other immune signaling molecules and is essential for the priming of immune cells, such as the T‐cell immunological synapses formation [55]. Moreover, the PML protein SUMOylation and its binding to SIM constitute the molecular basis for PML body formation [56].

Alterations in the extracellular environment of metabolic reprogramming, including oxidative stress and lactic acid accumulation [57], may trigger the activation of various PTMs on immune‐related proteins, especially SUMOylation, which dynamically responds to such external stimuli [58]. PTMs regulate the metabolic flexibility of various immune cells through fatty acid synthesis, glycolysis, mitochondrial oxidative metabolism, and mevalonate metabolism [59]. For instance, appropriate glycolysis, facilitated by TCR/CD28‐triggered PI3K‐AKT‐mTOR activation, promotes Treg cell proliferation, yet excessive glycolysis impairs its function. Given this correlation between PTM‐mediated metabolic flexibility and the altered immune environment, it is likely that PTMs may become conditionally essential for immune cells during an immune response [60].

Moreover, SUMOylation impacts the stability and plasticity of gene expression in Treg cells epigenetic regulation [7]. For instance, hypomethylation is significant for immune cells to maintain their identity and function in normal physiological conditions by stabilizing signature genes, such as Forkhead Box P3 (Foxp3), which is essential for their long‐term lineage commitment and immune homeostasis [61]. In contrast, the dysregulation of SUMO‐SIM interaction‐mediated crosstalk between SUMOylation and other PTMs, especially methylation and acetylation, enables some conserved regions of immune cells to be activated and rewritten, leading to transcriptional and/or epigenetic alterations [62].

### 2.4 Acetylation

Acetylation is a dynamic, highly specific, and reversible PTM that has a significant impact on nuclear transcription and metabolic homeostasis by counteracting the positive charge of lysine residues, ultimately altering protein electrostatic interactions and chromatin structure [63]. It significantly determines the activation level and immune tolerance status of immune cells by modulating chromatin accessibility and the activity of key signaling molecules, thereby achieving long‐term and stable immune regulation [64].

Acetylation denotes the process of covalently attaching an acetyl group to the N‐terminal α‐amino group (Nα‐acetylation), the hydroxyl group of tyrosine, serine, and threonine (O‐acetylation), or the ε‐amino group of lysine residue (Nε‐acetylation) of a protein, either enzymatically or non‐enzymatically [65]. Approximately 85% of human proteins undergo Nα‐acetylation and this process is irreversible [66]. The role of O‐acetylation in immune regulation requires further clarification. Immune‐related proteins primarily undergo Nε‐acetylation, and it is catalyzed by lysine acetyltransferase (KAT/HAT). However, deacetylation catalyzed by lysine deacetylases (KDACs/HDACs) removes the acetyl group [67]. This regulatory model utilizes the metabolic product acetyl‐CoA as a molecular bridge to directly translate the metabolic state of immune cell into signals that regulate proteins function. For example, the acetylation of glyceraldehyde‐3‐phosphate dehydrogenase can relieve its translational inhibition of IFN‐γ mRNA, thereby enhancing cellular immunity [68].

Nε‐acetylation can be further categorized into histone acetylation and non‐histone protein acetylation. Histone acetylation significantly enhances the transcriptional activation of immune‐related genes by relaxing the dense structure of chromatin, and ultimately regulates the activation phenotype of immune cells [69]. For example, histone acetylation determines whether dendritic cells (DCs) induce immune tolerance or initiate a strong immune response by regulating the expression of CD80/CD86 [70]. Non‐histone acetylation directly regulates the activity of key TFs, such as acetylation at Lys310 of the NF‐κB p65 subunit, which promotes its transcriptional ability and upregulates inflammatory factors [71].

### 2.5 Methylation

Methylation, as a kinetically slow and reversible PTM, can achieve finely graded regulation through site specificity and the degree of methylation. It can establish stable epigenetic marks via histone, DNA, and RNA modifications, determining the lineage fate of immune cells and long‐term immune tolerance [72]. It can also directly methylate key signaling molecules, precisely regulating the intensity of immune responses and inflammatory homeostasis [73].

Methylation refers to the process in which a methyl group is transferred from S‐adenosylmethionine (SAM) to specific amino acid residues, primarily lysine and arginine [74]. It is generally believed that methylation occurs at a slower rate than many other PTMs [75]. Demethylation is a reversible process catalyzed by demethylases that removes methyl groups from proteins. Lysine methylation is catalyzed by protein lysine methyltransferases (PKMTs) and can be monomethylation (Kme1), dimethylation (Kme2), and trimethylation (Kme3), which are associated with transcriptional silencing or activation [76]. Studies have shown that several protein arginine methyltransferases (PRMTs) that mediate arginine methylation are essential for the lymphoid and myeloid cell lineages establishment and maintenance [77]. For example, PRMT1 regulates interleukin‐6 (IL‐6) production in macrophages [78]. Furthermore, the absence of PRMT5 in CD4^+^ helper T‐cell subsets inhibits Th17 cell differentiation [79].

Methylation undertakes one of the most critical biological roles in histone modifications. Histone methylation denotes the addition of methyl groups to lysine or arginine side chains within histones. The modification sites and methylation levels on histone tails are associated with gene differential expression [73]. For example, monomethylation of histone H3 at lysine 4 (H3K4me1) is typically associated with enhancer function, whereas H3K4me3 is correlated with promoter activity [80]. H3K79me2 is crucial for cell cycle regulation, while H3K79me3 is associated with the Wnt signaling pathway [81]. Some histone methylation marks are repressive, including H3K9me3, H3K27me3, and H4K20me3, while others are activatory, including H3K4me3, H3K36me3, and H3K79me3 [82]. Numerous studies have shown that lineage‐specific differences in histone methylation exist on effector genes during CD4^+^ T‐cell differentiation. Specifically, the *IFNG* gene in Th1 cells and the *IL4* gene in Th2 cells both exhibit the transcriptionally permissive mark H3K4me3. Conversely, the *IL4* gene in Th1 cells and the *IFNG* gene in Th2 cells are modified by the transcriptionally repressive mark H3K27me3 [83]. In addition, methylation can directly target non‐histones, such as key immune signaling molecules. For example, PRMT6 upregulates NF‐κB by directly interacting with RelA/p65 and enhancing its nuclear translocation [84]. PRMT7 upregulates IFN production via catalyzing the Arg232 site methylation in MAVS, thereby preventing excessive inflammation [85].

### 2.6 Glycosylation

Glycosylation, a structurally intricate, abundant, and evolutionarily conserved form of PTM, primarily modifies secreted proteins (e.g., adhesion molecules, antibodies, and cytokines), as well as membrane‐bound proteins (e.g., membrane receptors), and is essential for the diversity and sophistication of immune‐related glycoprotein structures and relevant functions [86]. This complex glycosylation precisely regulates immune recognition, antigen presentation, immune effector mechanisms, receptor engagement, and activation processes.

Protein glycosylation is the process in which monosaccharide or polysaccharide chains are covalently linked protein residues via glycosyltransferases [87]. It includes N‐glycosylation, O‐glycosylation, C‐mannosylation, and the formation of glycosylphosphatidylinositol (GPI)‐anchored proteins, among which N‐glycosylation and O‐glycosylation are the two most common types [88]. N‐linked glycosylation is found on Asn, which forms the conserved Asn‐X‐Ser/Thr motif, where X represents any amino acid except proline. Subsequently, the N‐linked glycosylation is processed in the endoplasmic reticulum (ER) and acquires its final form in the Golgi apparatus [89]. N‐glycosylation can stabilize the folding and membrane expression of immune receptors, including T‐cell receptors (TCR), B‐cell receptors (BCR), and Toll‑like receptor (TLR), regulate antigen recognition and immune synapse formation, and it is the basis for lymphocyte activation [90]. O‐glycosylation is heterogeneous and complex, involving the addition of sugar chains to serine and threonine residues, and they have caused a large repertoire of carbohydrate structures present on almost every secreted and integral membrane protein [91]. O‐glycosylation participates in the migration, homing, and adhesion of immune cells, and regulates inflammatory infiltration and immune tolerance by modifying mucins and ligand molecules [92].

Moreover, galactosylation specifically refers to the glycosylation process that uses galactose as the core modification residue. Under the action of galactosyltransferases, galactose residues are attached to the terminal positions of oligosaccharide chains on glycoproteins and glycolipids [93]. As a key type responsible for terminal modifications in the glycosylation network, it exerts a significant biological effect in immune regulation and immune cell differentiation. For example, galactosylation can significantly alter the spatial conformation of the IgG Fc region, promoting the hexamerization of IgG molecules, ultimately enhancing the antibody's affinity for the complement component C1q and strongly activating the classical complement pathway [94].

### 2.7 Other PTMs

In addition, besides the aforementioned modifications, other PTMs including S‐Nitrosylation, lysine succinylation, lactylation, lysine malonylation (K‐Mal), and citrullination also regulate immune cell functions. Among them, S‐nitrosylation is a PTM that entails the attachment of a nitroso group to the cysteine thiol, generating S‑nitrosothiol, participating in the regulation of protein stability, immune‐related TFs, as well as the growth and apoptosis of immune cells [95]. Lysine succinylation modulates the growth and metastasis of tumor cells, and correlates with DNA damage response (DDR) and immunity [96]. Lactylation can convert the metabolic state of cells into regulatory signals for gene expression, promoting the differentiation of immune cells such as macrophages into pro‐inflammatory or reparative states, thereby playing a central immunoregulatory role in infections, wound repair, and inflammatory diseases [97]. K‐Mal, an essential yet incompletely PTM, in which a malonyl group is transferred to the ε‐amino group of lysine, altering the functional characteristics of substrate proteins. It has been associated with various metabolic pathways, including gluconeogenesis, glycolysis, the urea cycle, and fatty acid β‐oxidation [98]. Citrullination converts the amino acid arginine to citrulline catalyzed by the enzyme peptidyl arginine deiminase peptidylarginine deiminase (PAD) and participates in innate immune defense and inflammatory responses [99].

## 3 The Function of PTMs in Innate Immunity

**FIGURE 2:**
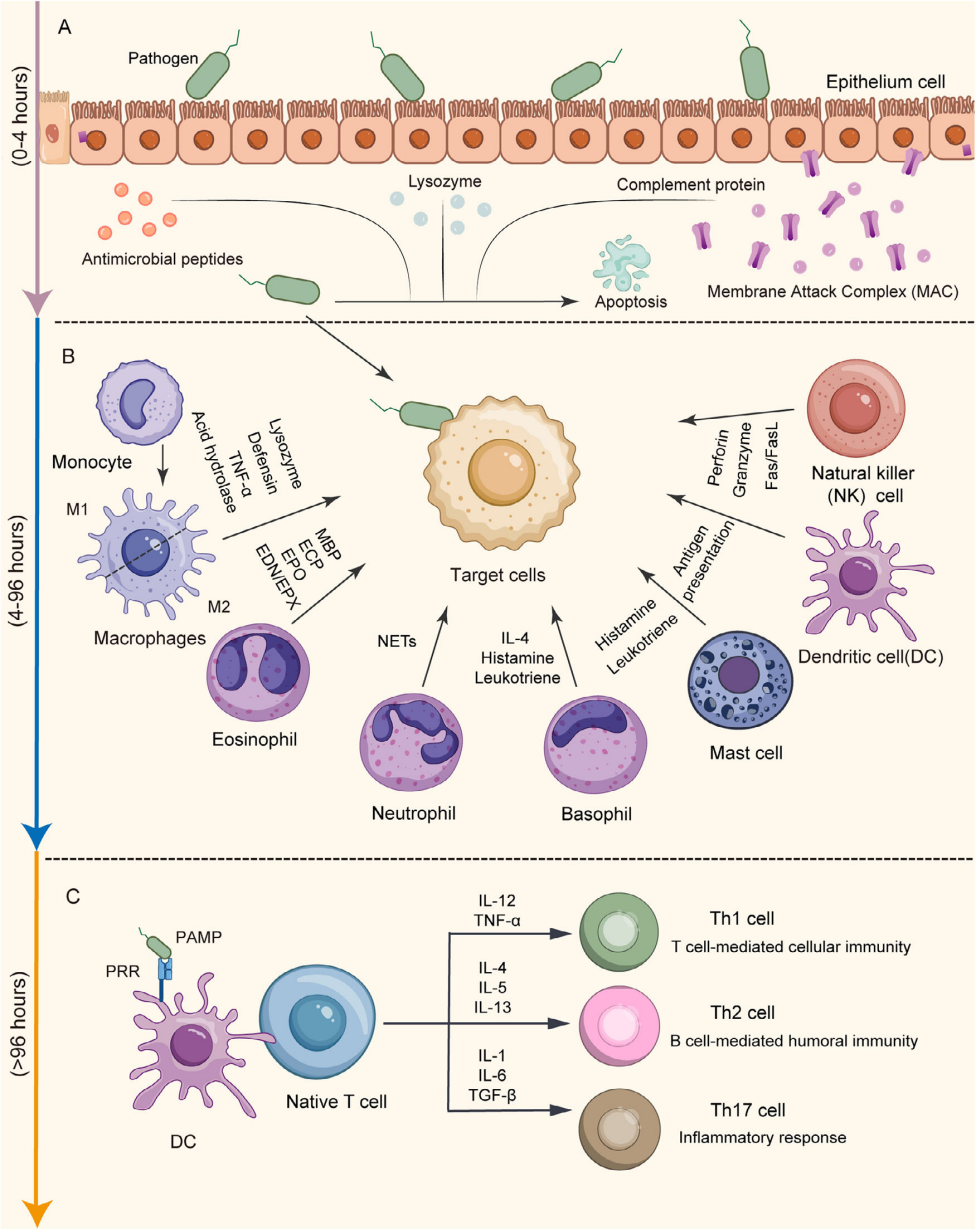
The entire process of innate immunity. (A) In the immediate defense phase (0–4 h), pathogens breach the epithelial barrier, and the first line of innate immune defense is mobilized, comprising antimicrobial peptides, lysozymes, and the complement cascade, which act directly to kill pathogens. (B) In the early induced responses (4–96 h), innate immune cells such as macrophages, neutrophils, NK cells, and DCs are recruited and activated, clearing pathogens through mechanisms like phagocytosis, cytokine release (e.g., TNF‐α and IL‐4), and neutrophil extracellular traps (NETs). (C) In the instruction of adaptive immunity phase (>96 h), DCs initiate the adaptive immune response through antigen presentation to naive T cells. The differentiation of naive T cells into Th1, Th2, or Th17 cells is directed by the cytokines produced by DCs, such as IL‐12, IL‐4, and IL‐6, which mediate cellular immunity, humoral immunity, and inflammatory responses, respectively, thereby achieving pathogen‐specific clearance. IL, interleukin; PAMP, pathogen‐associated molecular pattern; PRR, pattern recognition receptor; TNF‐α, tumor necrosis factor‐α; TGF‐β, transforming growth factor‐β.

The innate immune response is inherently present in the organism and lacks memory, providing a crucial first line of defense for the host to rapidly and non‐specifically recognize and eliminate pathogens through dynamic and intricate interactions among its numerous immune cellular and molecular components [100]. A series of PTMs act in a coordinated fashion to influence the three response phases of innate immunity, including the immediate defense, the early induced responses, and the instruction of adaptive immunity, thereby constituting the core barrier of innate immunity (Figure 2). PTMs precisely mediate innate immune responses by regulating anatomical barriers, innate immune cells, and innate immune molecules (e.g., complement proteins and cytokines), maintaining immune homeostasis and preventing excessive inflammatory damage [101].

### 3.1 The Immediate Defense

This stage occurs within 0–4 h after infection, during which the body relies on physical and chemical barriers to block pathogen invasion, including skin, mucous membranes, and their secretions [102]. By altering protein conformation, stability, and PPIs both within and between innate immune cells, PTMs, especially glycosylation, may change the properties and biological roles of these cells [103]. If pathogens breach the tissue barriers, certain innate immune mechanisms are immediately activated. These immediate defenses include several pre‐existing soluble molecules found in extracellular fluid, blood, and epithelial secretions, which can either kill pathogens or weaken their effects [104]. Lysozyme begins to break down bacterial cell envelopes, and the glycosylation of lysozyme can significantly enhance its stability by inhibiting proteolytic degradation, thereby continuously exerting its antibacterial effect [105]. While antimicrobial peptides can directly disrupt bacterial cell membranes, the complement system is activated through the alternative pathway, leading to pathogen lysis or phagocytosis [106].

### 3.2 The Early Induced Responses

This stage occurs within 4–96 h after infection. If the immediate defense mechanism fails, innate immune cells will be activated through pattern recognition receptors (PRRs), which can detect molecules specific to microbes, known as pathogen‐associated molecular patterns (PAMPs), or molecules released by damaged or dying cells called damage‐associated molecular patterns (DAMPs) [107]. Various PTMs can precisely regulate the functions of activated innate immune cells by modulating relevant TFs and cytokines, including granulocytes, monocytes/macrophages, DCs, and innate lymphoid cells (ILCs), ultimately initiating multiple effector mechanisms to clear infections [64].

#### 3.2.1 The Recognition Stage

The characteristic of innate immunity in recognizing pathogens lies in its ability to accurately distinguish the host components from structures unique to pathogens, which are only expressed in microorganisms. This recognition process is mediated by various PRRs expressed by host cells. Phosphorylation, polyubiquitination, and SUMOylation can achieve precise recognition of pathogens by dynamically modulating the activation, location, and stability of PRRs, adaptor proteins, and transcription‐related factors [108].

**FIGURE 3:**
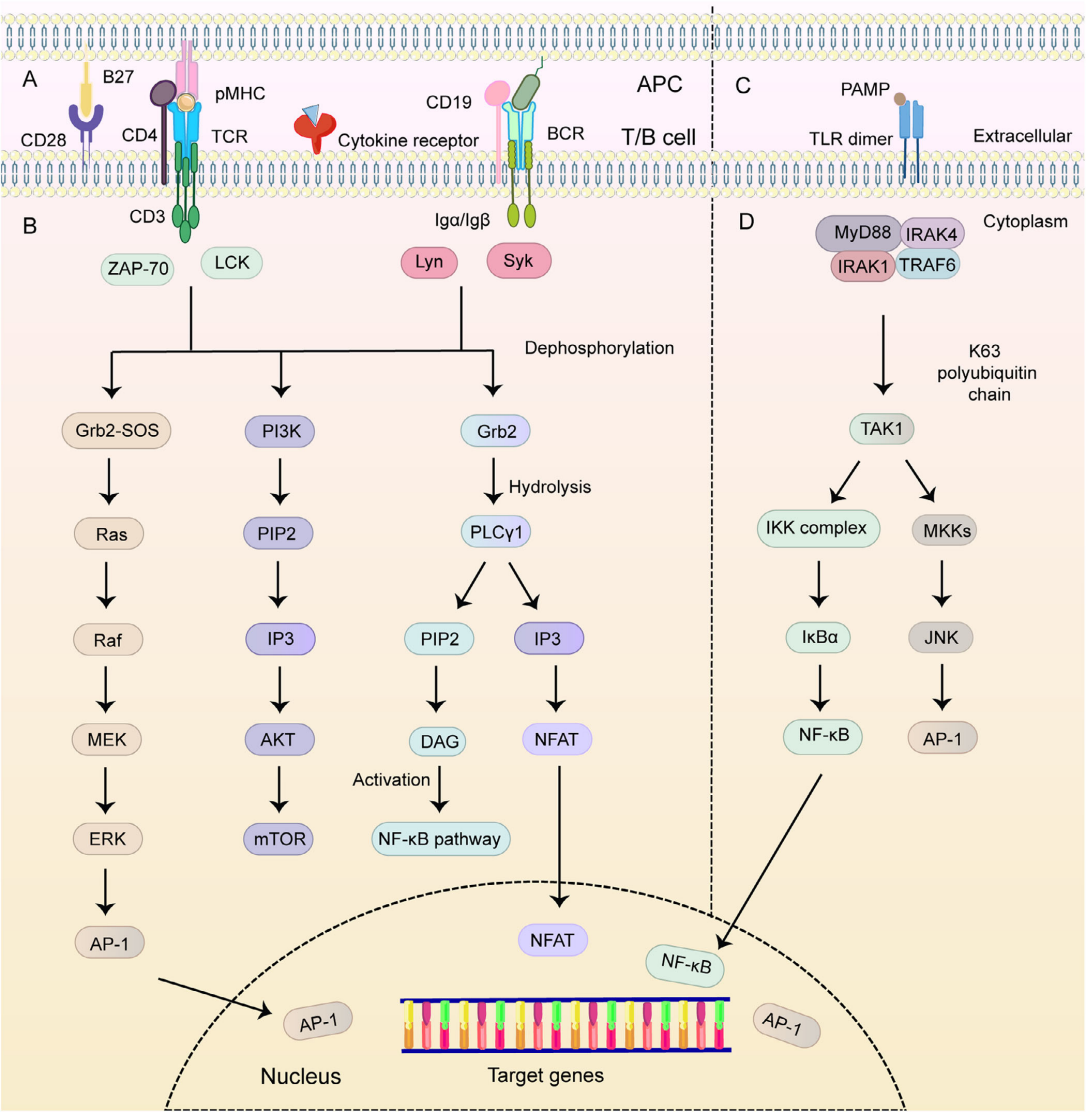
Signal transduction pathways mediated by T/B cell receptors and TLRs. (A) The TCR complex on T cells recognizes pMHC complexes presented by APCs. The BCR complex on B cells binds to antigens. TLRs on innate immune cells recognize PAMPs. (B) Upon TCR ligation, kinases Lck and ZAP‐70 are activated, triggering three major signaling branches, the Ras‐MAPK pathway, the PI3K‐AKT‐mTOR pathway, and the PLCγ1‐mediated pathway. Similarly, BCR activation via Lyn and Syk initiates analogous pathways. (C and D) Upon PAMP recognition, TLR dimers recruit the adaptor MyD88, leading to activation of IRAK/TRAF6 and TAK1. This triggers NF‐κB signaling and activates MKK/JNK to induce AP‐1 activation. Collectively, AP‐1, NFAT, and NF‐κB translocate to the cell nucleus, where they upregulate immune‐related target genes. AP‐1, activator protein 1; ERK, extracellular signal‐regulated kinase; Grb2, growth factor receptor‐bound protein 2; IKK, IκB kinase; IP3, inositol 1,4,5‐trisphosphate; IRAK, interleukin‐1 receptor‐associated kinase; JNK, c‐Jun N‐terminal kinase; LCK, lymphocyte‐specific protein tyrosine kinase; MEK, MAPK/ERK kinase; mTOR, mechanistic target of rapamycin; NF‐κB, nuclear factor kappa B; NFAT, nuclear factor of activated T cells; PAMPs, pathogen‐associated molecular patterns; PI3K, phosphoinositide 3‐kinase; PLCγ1, phospholipase C gamma 1; PIP2, phosphatidylinositol 4,5‐bisphosphate; pMHC, peptide‐MHC; TAK1, transforming growth factor‐β‐activated kinase 1; TLR, Toll‐like receptor; TRAF, TNF receptor‐associated factor; ZAP‐70, zeta chain of T cell receptor‐associated protein kinase 70.

PPRs include TLRs, nucleotide‐binding oligomerization domain‐like receptors (NLRs), C‐type lectin receptors (CLRs), and retinoic acid‐inducible gene I‐like receptors (RLRs) [109]. PAMPs are highly conserved molecular structures present in bacteria, viruses, fungi, and parasites, while DAMPs can send tissue damage signals and promote immune responses [110]. Different PRRs can detect conserved molecular signatures on diverse pathogens. TLRs and NLRs can rapidly activate downstream adaptor proteins upon ligand binding, which in turn activate kinases like IRAK and TAK1 (Figure 3) [111]. These kinases catalyze the phosphorylation of downstream signaling molecules, ultimately activating TFs including NF‐κB, MAPK, and IRF. After phosphorylation, NF‐κB can translocate into the nucleus to induce expression of pro‐inflammatory cytokines and chemokines genes [112]. Phosphorylation of IRF3/7 can drive the synthesis of IFN‐α and IFN‐β, laying the foundation for the subsequent effector phase. Meanwhile, phosphorylation of PRRs themselves can also enhance their ligand‐binding ability, amplifying recognition signals [112]. In the TLR pathway, K63‐linked polyubiquitination of tumor necrosis factor (TNF) receptor‐associated factor6 (TRAF6) and TRAF3 is required for NF‐κB and IRF3 activation. K48‐linked polyubiquitination can target IκB for proteasomal destruction, thereby allowing NF‐κB nuclear translocation [113].

#### 3.2.2 The Granulocytes

Granulocytes recognize, engulf, and eliminate microbial pathogens by oxidative mechanisms, lysozyme, collagenase, and elastase, serving as the dominant effector cells in the early innate immune response and the acute inflammatory phase [114]. The activation and effector functions of granulocytes are highly dependent on PTMs. Modifications affect the intensity of immune responses and the process of inflammation by finely regulating intracellular signaling molecules and enzyme activities.

Granulocytes include neutrophils, eosinophils, basophils, and mast cells. When pathogens cause local infections, neutrophils, which have strong chemotactic activity, resist pathogens through phagocytosis, extracellular trap formation, and degranulation [115]. Neutrophil activation includes priming characterized by enhancers marked by histone H3K27ac and H3K4me1, and direct activation characterized by a significant increase in H3K4me3 marking transcription start site regions [116]. PAD4 is a nuclear neutrophil enzyme that can catalyze the citrullination of histone H3 and mediate the formation of neutrophil extracellular traps at wound sites [117]. Eosinophils kill parasites and pathogenic microorganisms by releasing toxic effector molecules [118]. IL‐5 can stimulate eosinophil activation and induce degranulation. Membrane‐associated RING‐CH‐type finger protein (MARCH) binds to the IL‐5Rα and mediates polyubiquitination at Lys379 and Lys383 sites, followed by its degradation in lysosomes, thereby preventing excessive signal activation [119]. Basophils and mast cells enhance the early inflammatory process by releasing leukotrienes, histamine, and eosinophil chemotactic factors [120].

#### 3.2.3 The Monocytes/Macrophages

Monocytes circulate in the peripheral blood, and they can differentiate into macrophages and perform functions including phagocytosing pathogens, presenting antigens, and promoting cytokine secretion, and can also produce cytotoxic factors, including TNF‐α, IL‐6, IL‐1, IP‐10, macrophage inflammatory protein 1 (MIP‐1), and monocyte chemoattractant protein 1 (MCP‐1), ultimately involved in anti‐tumor immunity [121]. Ubiquitination, deSUMOylation, and acetylation regulate macrophages phagocytic bactericidal function by affecting macrophage polarization and associated signaling molecules.

Macrophages can polarize into M1 or M2 subtypes, among which M1 macrophages possess pro‑inflammatory functions and drive anti‑tumor immunity, whereas M2 macrophages exhibit immunosuppressive properties and facilitate tumor progression [122]. Research has already shown that succinate can activate HIFα and induce the expression of IL‐1 in M1 macrophages [123]. Kruppel‐like factor4 (KLF4) is a TF that regulates macrophage polarization, and macrophages with a ubiquitination‐deficient Lys278 site of KLF4 can upregulate the expression of M1 macrophage‐related genes in tumor cells. Under stimulation by LPS, SENP1 enters the mitochondria to deSUMOylate Sirt3, thereby activating Sirt3 and inducing macrophage polarization toward M2 [124]. Moreover, the STAT6 acetylation influences tumor‐associated macrophages capability to destroy tumor cells. STAT6 generally drives macrophage polarization toward M2, thereby suppressing immune responses. However, Lys383 site acetylation in STAT6 can inhibit M2 polarization, thus enhancing the anti‐tumor ability of macrophages [125].

#### 3.2.4 The DCs

DCs, as the most potent antigen‐presenting cells (APCs), are essential mediators of the innate immune system by recognizing, capturing, and processing exogenous antigens, and inducing T‐cell activation and proliferation. Activated DCs can produce IFNs that mediate antiviral innate immune responses [126]. Succinylation is important in regulating the phenotype of DCs. The pivotal metabolic enzyme succinyl‐CoA synthetase β subunit Suclg2 promotes the reprogramming of the pro‐inflammatory state of mature DCs into a tolerogenic phenotype by inhibiting the activation of the NF‐κB signaling pathway [127]. SUMOylation plays a negative role in modulating the anti‐microbial capacity of DCs. The deficiency of SUMOylation in DCs can markedly elevate the expression level of IFN‐β by modifying distant regions downstream of the Ifnb1 promoter, ultimately enhancing their resistance to bacterial infection [128].

#### 3.2.5 The ILCs

ILCs include ILC1, ILC2, ILC3, natural killer (NK) cells, and lymphoid tissue inducer (LTi) cells, which lack surface markers of other immune cell lineages and are mainly involved in organizing homeostasis and inflammation [129]. NK cells are the only ILCs that have direct killing functions, exerting a crucial function in innate immunity against viruses and cytoplasmic pathogens, as well as in antibody‐dependent cell‐mediated cytotoxicity (ADCC). In addition, NK cells contain granules loaded with perforin and granzyme, which can induce lysis or apoptosis in target cells, and also express PRRs [130]. As a unique form of glycosylation, sialylation can regulate NK cell activity by balancing activation and suppressive effects. For example, Siglec‐7 and Siglec‐9 are negative receptors on NK cell surfaces that interact with sialylated molecules. When these receptors bind to sialylated glycan chains on target cells, inhibitory signals are transmitted to the NK cells, thereby decreasing their potential to kill target cells [131]. Moreover, cytokines increase sialylation levels on the surface of NK cells, thereby possibly modifying receptor functions and the general reactivity of NK cells [132].

### 3.3 The Instruction of Adaptive Immunity

Innate immunity can further accomplish the clearance of pathogens by precisely initiating and tightly regulating the activation and effector processes of adaptive immune responses, which occurs 96 h after infection [8]. The interaction of PTMs in activation‐specific proteins and/or in the initiation of key innate immune responses occurs at both microscopic and macroscopic levels. Microscopically, a particular PTM depends on other PTMs for its recruitment, initiation, and even activation. Macroscopically, given the dynamic and mutually responsive nature of immune processes, all PTMs ultimately act in a coordinated fashion to collectively participate in and influence the entire immune response, including T‐cell‐mediated cellular immunity, B‐cell‐mediated humoral immunity, innate immunity, and the crosstalk among them [108].

Innate immunity plays a pivotal hub that initiates adaptive immune responses, among which APCs are the core bridge connecting innate and adaptive immunity. APCs employ PRRs to detect and internalize pathogens and present peptide‐MHC (pMHC) complexes on the surface for recognition. Concurrently, activated APCs significantly upregulate co‐stimulatory molecules, and these two signals act collaboratively to initiate an adaptive immune response [133]. Glycosylation not only protects MHC II from premature proteolysis in non‐acidic vesicles to guarantee that antigenic peptides can be bound by MHC II groove but also ensures high‐affinity binding of the complex molecules to antigenic peptides and efficient recognition by TCRs [134]. Innate immunity also regulates the effector functions of adaptive immune responses by producing different cytokines. In cellular immunity, cytokines including IL‐2 secreted by activated Th1 cells activate macrophages, enhancing their ability to clear target antigens. The acetylation at Lys696 of STAT5 enhances the transcription of the *IL‐2* gene [135]. In humoral immunity, macrophages, NK cells, and complement effectively eliminate pathogens through opsonization, ADCC, and complement‐mediated lysis.

## 4 The Function of PTMs in T Cell‐Mediated Cellular Immunity

**FIGURE 4:**
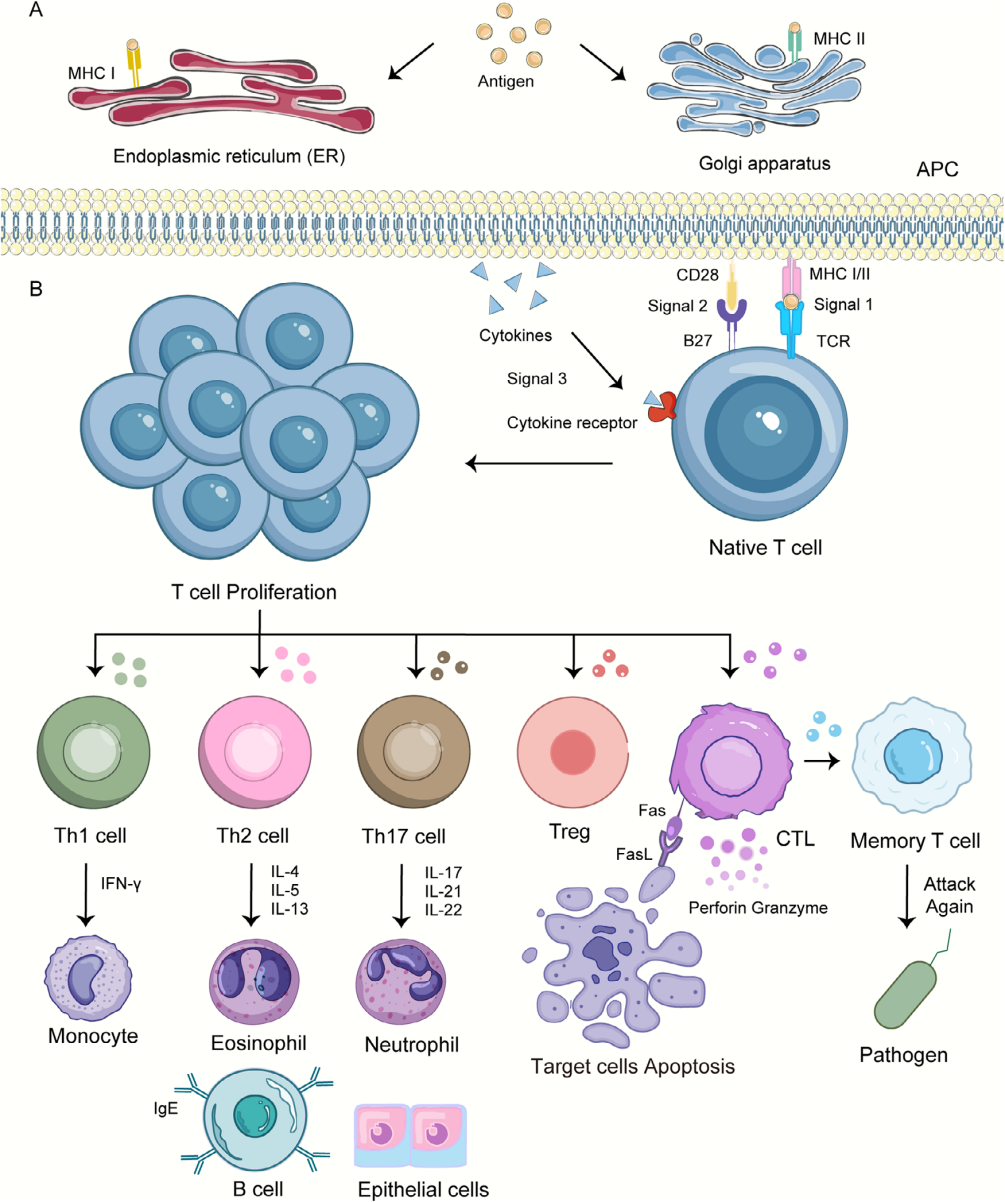
The entire process of T‐cell‐mediated cellular Immunity. (A) APCs capture and process pathogen antigens, and then present antigen peptides to naive T cells through MHC molecules. (B) Th1 cells, driven by IL‐2, produce IFN‐γ, which activates monocytes and macrophages, promote the differentiation of CTLs, and thereby orchestrate cellular immunity. Th2 cells, which develop in response to IL‐4, secrete IL‐4, IL‐5, and IL‐13. These cytokines stimulate eosinophils and B cells, supporting B‐cell proliferation, differentiation, and antibody class switching, thus mediating humoral immunity and defense against parasites. Th17 cells are characterized by the production of IL‑17 and related cytokines. They recruit and activate neutrophils, contribute to the elimination of extracellular bacteria, and drive inflammatory responses. Their dysregulation is closely linked to the pathogenesis of autoimmune diseases. Tregs do not directly participate in effector responses; instead, they suppress immune activation and help maintain peripheral tolerance, preventing excessive tissue damage. CTLs induce apoptosis of target cells by releasing perforin, granzymes, and expressing FasL, directly eliminating infected or abnormal cells. After the formation of memory T cells, they can be rapidly activated upon re‐exposure to pathogens, mediating a secondary immune response. APC, antigen‐presenting cells; CTL, cytotoxic T lymphocyte; Treg, regulatory T cell.

T‐cell‐mediated cellular immunity is a highly specific adaptive process that eliminates intracellular pathogens and abnormal somatic cells through effector cells and molecules. It is a core element of the adaptive immune system, encompassing the recognition of antigens, the activation of T cells, the elimination of antigen, the contraction phase, and the immune memory (Figure 4). Notably, to respond rapidly to changes in surrounding antigen or pathogens, T‐cell‐mediated cellular immunity may harness PTMs to regulate immune‐related protein structure, conformation, activity, and PPIs, as these modifications occur faster than de novo protein synthesis. PTMs, which may intrinsically evade genetic confinement, finely regulate cellular immune responses by dynamically modulating immune‐related proteins.

### 4.1 The Recognition of Antigens

As the initial phase of cellular immune response, the core feature of antigen recognition is that specific TCR on T cells recognize the antigen pMHC on the surface of APCs [136]. During antigen recognition, PTMs indispensably regulate the functions of TCR, MHC molecules, and related regulatory factors to ensure the precision and efficiency of the immune response [137].

MHC I and MHC II initiate immune responses against pathogens and malignantly transformed cells. The presentation of MHC I molecules mainly involves proteins produced inside the cell. In contrast, peptides presented by MHC II mainly come from extracellular pathogens and other antigens [138]. Glycosylation occurs on the TCR αβ chains and the surface of MHC II, which are significant for T‐cell activation. TCR N‑glycans primarily ensure proper cell‑surface localization and molecular orientation of the receptor [134]. Removing N‑glycans on MHC II impairs live APC‑mediated glycoantigen presentation, nearly abrogates their binding to recombinant MHC II in vitro, and strongly suppresses glycoantigen‑driven T‐cell activation and recognition, leading to dysregulation of intestinal immune balance (Figure 3) [139].

### 4.2 The Activation of T Cells

After antigen recognition, T‐cell activation is the process of gradually initiating gene expression, secreting cytokines, and ultimately achieving self‐activation and proliferation [140]. Initial T cells require TCR recognition of antigen, co‐stimulatory signal, and cytokines to be fully activated and proliferate. In contrast, the activation of memory T cells can more rapidly promote the production of cytokines [141]. PTMs rapidly and reversibly convert external antigen stimulation signals into intracellular molecular responses, ensuring that T cells can be activated at the appropriate time, including phosphorylation, ubiquitination, SUMOylation, and glycosylation.

The specific recognition of the pMHC complex by the TCR marks the beginning of the T‐cell activation process. This triggers rapid clustering of T‐cell receptor molecules, forming the so‐called immunological synapse [142]. After antigen recognition, the phosphorylation cascade driven by lymphocyte‐specific protein tyrosine kinase (Lck) is initiated. The TCR and CD28 signals cause the activation of the protein kinase cascade. It activates TFs to induce the expression of genes associated with T‐cell proliferation and survival [143]. Activated zeta chain of T‐cell receptor‐associated protein kinase 70 (ZAP‐70) molecules can be catalyzed for ubiquitination by Cbl. Meanwhile, ZAP‐70 itself also undergoes ubiquitination, which is regarded as a signal for its degradation or termination of signal transduction [144]. Nuclear factor of activated T cells (NFAT) regulates T‐cell activation and influences cytokine generation. SUMOylation regulates the localization of NFAT, enabling its recruitment to early promyelocytic leukemia bodies and interaction with HDAC. This inhibits IL‐2 expression, ultimately downregulating T‐cell activation [145]. Moreover, blocking SUMOylation at the Lys237 on JunB inhibits the expression of IL‐2 and IL‐4 in T cells [146].

### 4.3 The Elimination of Antigen

After successful activation, T cells enter a crucial phase characterized by rapid proliferation and differentiation, producing numerous effector cells and molecules that mediate antigen clearance [147]. During the subsequent antigen elimination stage, various PTMs profoundly influence T‐cell differentiation choices and effector function execution by finely regulating key TFs and the functional states of their active proteins. The antigen elimination process, driven by various PTMs, relies on a delicate balance among helper T cells (Th) and regulatory T cells (Tregs) derived from CD4^+^ T cells, as well as cytotoxic T lymphocytes (CTLs) derived from CD8^+^ T cells, to effectively defend against antigens while avoiding autoimmunity [148].

#### 4.3.1 Helper T Cells (Th)

Th cells mainly includes three functional subsets, Th1, Th2, and Th17. Through the specific functions and synergistic effects of each subset, jointly clear the intracellular pathogens [149]. Various PTMs control Th cell fate and effector activities by controlling the activity and stability of Th‐related key TFs and signaling molecules.

Under the influence of IL‐2 signaling, T cells differentiate into Th1 cells, which secrete IFN‐γ and IL‐2 [150]. In this process, EzH2 significantly hinders the differentiation of Th1 by promoting H3K27me3 levels of Th lineage TFs and suppressing the expression of cytokines [151]. T‐bet phosphorylation at Thr302 specifically inhibits IL‐2 and Th2 cytokine expression during Th1 differentiation by interacting with activated NFAT [152]. It is worth noting that the cytokines released by Th1 cells exert multiple bioactivities in immune response [153]. The activation of STAT5 in this pathway depends on tyrosine phosphorylation mediated by JAK [154]. Furthermore, IFN‐γ enhances the phagocytic activity of mononuclear macrophages. In this pathway, methylation occurring at Lys525 of STAT1 promotes the binding STAT1 stability in the nucleus, ensuring that macrophages receive sufficient activation signals to carry out phagocytic clearance [155].

IL‐4 signaling induces the generation of Th2 cells, which produce IL‐4, IL‐5, and IL‐9. GATA‐3 is significant for chromatin remodeling at Th2 cytokine gene loci by inducing histone acetylation, sustaining the production of IL through KRR acetylation, and timely inducing Th2 cytokines [156]. Moreover, the methylation at Arg261 GATA‐3 selectively coordinates IL‐5 gene transcription by specifically binding to HSP60 in the IL‐5 gene promoter [157]. Similar to Th1 cells, Th2 cells secrete various cytokines that have different immunological functions. After IL‐5 activates the JAK2‐STAT5 signaling pathway, the Ser1126 site in the intracellular tail of the CD11b is phosphorylated, which changes the conformation of CD11b, promoting eosinophil adhesion, chemotaxis, and phagocytic function against antigens [158]. After IL‐4 binds to its receptor, phosphorylation of STAT6 at Tyr641 allows STAT6 to translocate into the nucleus [159].

As a TF critical for driving Th17 differentiation, the SUMOylation of RORγt at Lys187 site promotes HDAC2 recruitment at IL‐17 promoter and downregulates IL‐17 [160]. RORγt undergoes acetylation at various sites, but the acetylated Lys69, Lys81, and Lys99 are deacetylated in Th17 cells, which is crucial for the IL‐17 production and IL‐2 suppression [161]. IL‐21 acts as an enhancer of Th17 cells generation and activates NK cells [162]. IL‐17 induces the production of pro‐inflammatory cytokines and pro‐inflammatory chemokines. In addition, RORγt can be degraded by the proteasome pathway through K48‐linked polyubiquitination mediated by Itch [163].

#### 4.3.2 Treg Cells

The role of Treg is not to enhance acute immune responses but rather to suppress them. They help keep immune balance, prevent self‑reactive responses, and limit excessive inflammation. The TFs finely regulate the function of Treg cells through various PTMs [164].

As one of the most core TFs for Treg, Foxp3 can be regulated by multiple PTMs. Phosphorylation that occur at Foxp3 Ser418 positively regulates Treg suppressive function. Foxp3 acetylation inhibits proteasome‐mediated degradation and enhances Foxp3‐mediated transcriptional suppression of IL‐2 production [165]. TRAF6 binds Foxp3 and promotes its K63 ubiquitination at Lys262, which ensures the proper localization of Foxp3 to the cell nucleus and promotes its transcriptional activity in Tregs. Moreover, Foxp3 methylation induced by PRMT1 at Arg48 and Arg51 enhances its expression and promotes the immunosuppressive activity of Treg cells [166].

Since SUMOylation is highly dynamic, it plays a critical role in enabling Treg cells to rapidly respond to internal and external stimuli, including cytokine stimulation, TCR signaling activation, metabolic stress, and microenvironmental changes such as hypoxia, acidosis, and inflammation [7]. Under IL‐2 signaling stimulation, SENP1 enhances the transcriptional activity of STAT5 by rapidly removing its SUMOylation, thereby upregulating Foxp3 expression and ultimately maintaining the normal function of Treg [167].

#### 4.3.3 CTLs

Naive CD8^+^ T cells can differentiate into CTLs upon recognition of MHC I complexes and appropriate co‐stimulatory signals. Once activated by a series of coordinated PTM events, they efficiently and specifically kill target cells that present the target antigens, such as those containing intracellular pathogens and tumor cells, through the release of perforin and granzymes. PTMs collaboratively regulate their differentiation and effector functions [168].

CTLs induce target cell apoptosis through two main pathways. Perforin and granzymes are exocytosed and cooperatively induce target cell apoptosis and directly destroy infected or malignant cells. Glycosylation is vital for the regulation of perforin function [169]. N‐linked glycosylation occurring at Asn549 on the C‐terminal of perforin in ER maintains perforin spatial conformation stability. Thus, it ensures its effective secretion to the immune synapse and normal execution of membrane perforation [170]. The second pathway involves the engagement and aggregation of target‐cell death receptors and their cognate ligands on the killer‐cell membrane, such as Fas /FasL, which results in classical caspase‐dependent apoptosis. Glycosylation at N184, N250, and N260 in FasL ectodomain might be modified under certain conditions but they do not alter receptor binding [171]. Glycosylation of FasL stabilizes its localization and expression levels on the cell membrane, ensuring that it can effectively recognize and bind to Fas receptors on the surface of target cells. S‐nitrosylation occurring at the Cys304 residue of the Fas receptor can mediate Fas aggregation and recruit it to lipid rafts, a key platform for Fas signaling complex assembly, ultimately causing an increase in FasL‐mediated apoptosis [172].

### 4.4 The Contraction Phase

After antigen is eliminated, the response of effector T cells declines. Most effector cells are eliminated through apoptosis during this process, which marks a contraction phase and is important for restoring immune homeostasis [173]. Phosphorylation and acetylation modifications are important for effector T cells clearance.

In the late phase of the immune response, Tregs are induced to exert immunosuppressive effects [174]. Simultaneously, activated T cells that have completed their effector function can trigger activation‐induced cell death (AICD) by upregulating Fas/FasL expression in order to limit excessive tissue damage and restore immune homeostasis [175]. Bim is considered a mediator of signaling pathways that can initiate apoptosis. Thr112 phosphorylation mediated by JNK can enhance its binding affinity to Bcl2 and promote its dissociation from microtubules, thereby increasing pro‐apoptotic activity, whereas Ser55, Ser65, and Ser73 phosphorylation mediated by ERK promotes Bim degradation via the UPS [176, 177]. Additionally, acetylation of p53 at Lys382 can bind to the promoters of apoptotic genes, promoting their transcription and accelerating the execution of apoptosis under certain conditions [178].

### 4.5 The Immune Memory

Most effector cells are eliminated through apoptosis during the contraction phase, with a subset differentiating into long‐lived memory cells. These memory cells can generate faster and stronger responses upon re‐encountering the antigen, a response called the secondary immune response, which is less dependent on antigen concentration and co‐stimulatory signals, and more sensitive to cytokines than the primary response [179]. This enables the rapid initiation of an immune response at lower antigen concentrations, achieving efficient pathogen clearance and long‐term immune protection.

The unique characteristics of the above‐mentioned secondary responses are tightly connected to the regulation of memory T cells by various PTMs. Differentiation into memory CD8^+^ T cells requires widespread metabolic reprogramming and epigenetic remodeling, accompanied by the expression of unique surface molecules and TFs, especially TCF1 [180]. Studies have shown that after K‐Mal Lys374 site in STAT6, TCF1 expression is increased, ultimately promoting memory T‐cell generation [181]. After viral infection, the promoter and enhancer regions of the IFN‐γ gene in CD8^+^ memory T cells retain H3 acetylation. Similarly, resting CD4^+^ memory cells also exhibit elevated levels of histone H3 acetylation at the corresponding cytokine gene loci [182]. Moreover, Akt decreases CD8^+^ T‐cell memory precursor cells expansion and differentiation into functional memory cells by phosphorylating Ezh2, ultimately causing increased effector differentiation, sacrificing T memory precursors [183].

## 5 The Function of PTMs in BCell‐Mediated Humoral Immunity

**FIGURE 5:**
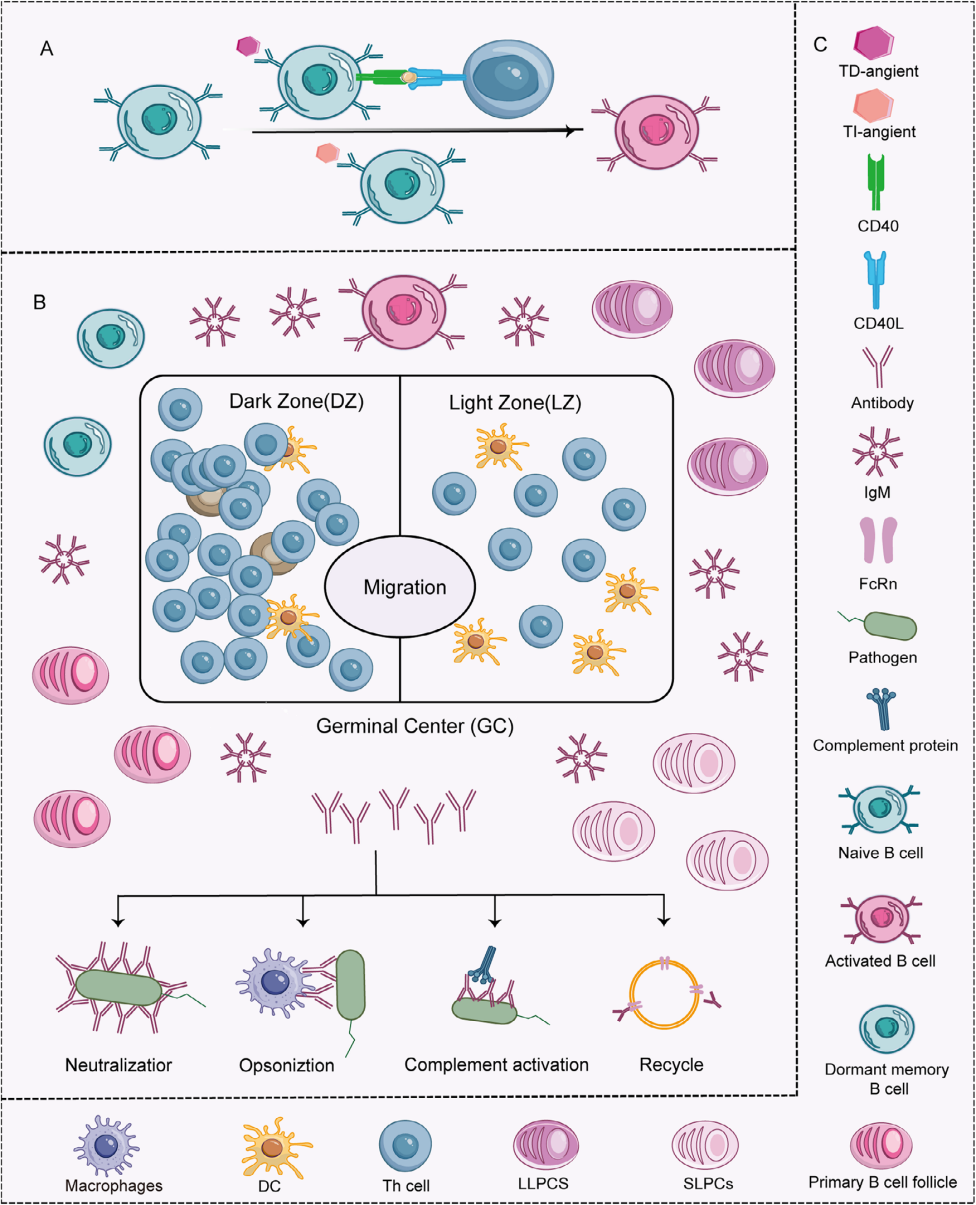
The entire process of B‐cell‐mediated humoral immunity. (A) Naive B cells can be activated by antigens through TD, CD40‐CD40L costimulation, or TI pathways, and differentiate into activated B cells. (B) Activated B cells enter the GC, undergo SHM, antibody affinity maturation, and antibody class switching. Subsequently, they proliferate in the DZ, migrate to the LZ for antigen selection, and finally differentiate into SLPCs, LLPCs, or memory B cells, and secrete IgM and other antibodies. Antibodies clear pathogens through neutralization, opsonization, and complement activation, and maintain antibody stability through FcRn‐mediated circulation. DZ, dark zone; FcRn, neonatal Fc receptor; GC, germinal center; IgM, immunoglobulin M; LLPCs, long‐lived plasma cells; LZ, light zone; SHM, somatic hypermutation; SLPCs, short‐lived plasma cells; TD, T‐cell‐dependent antigens; TI, T‐cell‐independent antigens;.

B cell‐mediated humoral immunity is an adaptive immune defense mechanism that specifically eliminates extracellular pathogens through antibody secretion and relies on memory cells and affinity maturation to achieve long‐term, efficient secondary responses [184]. Similar to cellular immunity, humoral immunity also includes the recognition of antigens, the activation of B cells, the elimination of antigen, the contraction phase, and the immune memory (Figure 5). The initiation of humoral immunity, signal transduction, and antibody effector functions all highly depend on the dynamic and coordinated regulation of PTMs. Various modifications finely alter the structure, stability, interactions, and subcellular localization of key molecules, coordinating core processes including BCR activation, antigen processing and presentation, as well as antibody production and class switching, thereby determining the strength, specificity, and immune homeostasis of humoral immune responses at the molecular levels [185].

### 5.1 The Recognition of Antigens

Unlike T cells that recognize processed peptides, mature B cells express BCR on surface that recognizes the native form of their corresponding antigen, which occurs in secondary lymphoid organs [186]. PTMs ensure the accuracy of the antigen recognition process by regulating key proteins. The surface of the BCR exhibits extensive and highly conserved N‐linked glycosylation. These glycans maintain the three‐dimensional structural stability of the BCR by increasing molecular rigidity, thereby preventing it from obscuring the antigen‐binding site. Fucosylation can alter the receptor conformation and affect its binding affinity with ligands, thereby participating in the regulation of BCR recognition of antigens [187]. FBXO11, as a member of the E3 ligase complex of CIITA, regulates CIITA protein level by ubiquitination of lysine residues. The degradation of CIITA significantly decreases the expression levels of MHC II and inhibits the antigen‐presenting function of MHC II [188]. In activated plasmacytoid DC (pDC), MARCH1, an E3 ligase, mediates ubiquitin tagging of MHC II and CD86, which promotes their internalization and lysosomal breakdown [189].

### 5.2 The Activation of B Cells

The activation process of B cells varies depending on the type of antigen. Thymus‐dependent (TD) antigen activation requires the specific BCR signal after recognizing an antigen and the CD40L–CD40 interaction [190]. In contrast, TI‐antigens activate B cells by binding to BCRs and mitogen receptors or by directly cross‐linking BCRs. Various PTMs finely regulate the transmission and amplification of activation signals by modulating BCR structural stability, antigen‐binding ability, and downstream signal transduction (Figure 3).

Protein phosphorylation is the core molecular mechanism that initiates and precisely regulates the BCR signaling cascade. When BCR recognizes and binds a multivalent antigen, the activation signals are transmitted by the Igα/Igβ (CD79a/b) heterodimer. Membrane‐proximal Src family tyrosine kinases are activated through autophosphorylation, and then phosphorylate the conserved tyrosines on the immunoreceptor tyrosine‐based activation motifs (ITAMs) in the cytoplasmic domains of the Igα/Igβ heterodimer [191]. Recruitment of spleen tyrosine kinase (Syk) to ITAMs bearing double phosphorylation undergoes complete activation by means of phosphorylation of essential tyrosine residues located in its kinase domain [192]. Syk is recruited to the doubly phosphorylated ITAMs and fully activated through phosphorylation of key tyrosine residues in its kinase domain. The activated Syk then phosphorylates multiple central adaptor proteins, particularly B cell linker protein (Blnk) and B cell adaptor protein (Bcap), forming a multi‐molecular signaling complex [193]. During TD antigen activation process, K63‐linked ubiquitination catalyzed by TRAF6 serves as a signal scaffold to promote the assembly of kinase complexes and amplify phosphorylation cascades. Meanwhile, K48‐linked ubiquitination negatively regulates A20 via proteasomal degradation, relieving signal inhibition [194]. CD40, CD80, and CD86 undergo N‐glycosylation modifications, maintain stable membrane localization conformations, and facilitate stable adhesion and cooperative signal transmission between T and B cells through immune synapse formation, thereby ensuring complete activation of B cells [195].

### 5.3 The Elimination of Antigen

Fully activated B cells rapidly enter the cell cycle under the influence of cytokines, proliferating and differentiating extensively to form plasma cells (PCs) and regulatory B cells (Bregs). Various PTMs finely regulate TFs, signaling molecules, and antibodies, not only enabling PCs to continuously secrete specific antibodies to eliminate pathogens through antigen neutralization, opsonization, and complement activation, but also allowing Bregs to produce anti‐inflammatory cytokines to downregulate immune responses, thereby ensuring the balance of immune responses [196].

#### 5.3.1 The PCs

Activated B cells mainly differentiate along two distinct pathways. One pathway leads to short‐lived plasma cells (SLPCs) differentiation and early serum antibodies production. The SLPCs mainly produce low‐affinity IgM antibodies, ultimately driving rapid primary defense [197]. Phosphorylated mTORC1, a serine/threonine kinase located in the ER and Golgi apparatus, can modulate ER transmembrane proteins, reducing ER stress and promoting the formation of SLPCs [198].

Another differentiation pathway causes long‐lived plasma cells (LLPCs) differentiation. Cytidine deaminase (AID) precise expression and localization are necessary prerequisites for the normal progression of somatic hypermutation (SHM), which is strictly regulated by epigenetic modifications. H3K9ac and H3K4me3 can promote transcription of immunoglobulin variable region genes targeted by AID and genes related to B‐cell proliferation and differentiation [199]. Meanwhile, phosphorylation of STAT3 and STAT6 activated by cytokine enters the nucleus and precisely regulates DNA recombination and transcription, ultimately achieving antibody class switching [200].

Antibodies contribute to the defense in various ways, including neutralization, opsonization, and activate the complement system [201]. N‐glycosylation is essential for proper antibody folding, oligomer assembly, and secretion efficiency [202]. The IgG Fc fragment contains a conserved N‐glycosylation site at Asn297. IgA also contains different N‐glycosylation sites. The glycan at Asn263 can stabilize monomeric IgA without affecting its ability to bind FcαR1. The human IgA2 subtype also has additional glycosylation sites at Asn166 in the Cα1 domain and Asn337 in the Cα2 domain [202, 203]. The glycans linked to these sites can not only neutralize viruses but also induce pro‐inflammatory responses in macrophages and neutrophils.

#### 5.3.2 Bregs

Breg cells, which secrete IL‐35, transforming growth factor‐β (TGF‐β), and IL‐10, are essential for simple contact hypersensitivity and complex systemic autoimmune diseases [204]. The antigen presentation functions of macrophages, inflammatory T cells, and B cells are all negatively regulated by IL‐35. TGF‐β can induce immature DCs to acquire immunotolerant properties. IL‐10 is a vital immunosuppressive factor that can inhibit CTLs function in TME and promote immune evasion [205]. SENP7 is a deSUMOylase that can activate SIRT1 by removing SUMO modifications on SIRT1. Activated SIRT1 further deacetylates STAT3, ultimately promoting IL‐10 expression [206].

### 5.4 The Contraction Phase

After antibodies complete their defensive function, the body recycles or removes antibodies through a series of finely regulated mechanisms to prevent excessive immune responses. PTMs can significantly regulate the processes, including antibody circulation, intracellular catabolism after fluid phase endocytosis, antigen‐mediated clearance, and the formation of anti‐therapeutic antibodies (ATA) by altering antibody structure, stability, and receptor binding ability [207].

Most intact IgG antibodies can be recovered. In acidic endosomal environments, the neonatal Fc receptor (FcRn) binds to IgG through protonated histidine residues, promoting receptor‐mediated recycling and transcytosis. In contrast, at neutral pH, deprotonation of these histidines induces IgG release, ensuring its efficient return to the circulatory system and preventing lysosomal degradation [208]. Glycosylation on the Fab fragment usually enhances IgG affinity for antigens and ultimately affects recycling efficiency. For example, the glycan attached to Asn58 of the IgG heavy chain can increase the antibody affinity by 10‐fold, while oligosaccharides at Asn54 can hinder IgG binding to antigens [195]. In addition, phosphorylation affects the intracellular clearance efficiency of antibodies by modulating the activity of intracellular pathway proteins, including those involved in antibody internalization and lysosomal degradation [209].

### 5.5 The Immune Memory

Humoral immune memory is mainly composed of memory B cells and LLPCs. These cells persist long‐term and rapidly proliferate and differentiate into PCs upon encountering the same or similar pathogens again, producing a large amount of specific antibodies [210]. Phosphorylation, deubiquitination, and epigenetic modifications jointly regulate memory B‐cell homeostasis.

In the resting state of memory B cells, cytokines such as IL‐7 and IL‐15 continuously stimulate their surface receptors, activating downstream signaling molecules Akt and STAT5. Phosphorylated Akt and STAT5 then shuttle into the nucleus to trigger the transcription of anti‐apoptotic genes, providing signals for memory B cells survival [154]. Meanwhile, the anti‐apoptotic protein Bcl2 avoids proteasomal degradation through deubiquitination, maintaining its protein stability [211]. Additionally, histone epigenetic modifications (such as H3K4me3 and H3K9ac) maintain chromatin in an open state, ensuring that memory B cells can stably express memory‐related genes even during the resting phase [212], thus preserving their cellular identity and functional characteristics. These modifications work together to ensure the long‐term survival of memory B cells, allowing them to rapidly activate and initiate an efficient secondary humoral immune response upon re‐encountering the same antigen.

## 6 PTMs in Immune‐Related Diseases

PTMs are critical regulators that dynamically modify the structure, stability, localization, and function of immune‐related proteins, thereby controlling the overall immune regulatory machinery. However, dysregulation of PTMs perturbs immune homeostasis, compromises immune tolerance, and upsets the homeostasis of pro‐inflammatory and anti‐inflammatory responses by affecting diverse aspects of immune cell development, activation, and differentiation, immune signal transduction, and effector functions [213, 214]. This is strongly associated with the onset and advancement of diverse immune‐related disorders, including cancer [215], autoimmune diseases [10], and infectious diseases [11].

### 6.1 Cancer

PTMs are vital for the activation of immune cells, signal transduction, and immune responses, but it is also commonplace in human cancers. PTMs can participate in regulating multiple hallmark features of cancer, including sustained proliferation, growth inhibition suppression, apoptosis resistance, replicative immortality, angiogenesis, and invasion [216]. Whether influencing cancer directly by modulating immune checkpoints (ICs), or indirectly by reshaping the TME, PTMs profoundly affect cancer cell immunogenicity, thereby influencing their vulnerability to immune recognition and destruction by the immune system [217].

#### 6.1.1 Modulation of the ICs by PTMs

**FIGURE 6:**
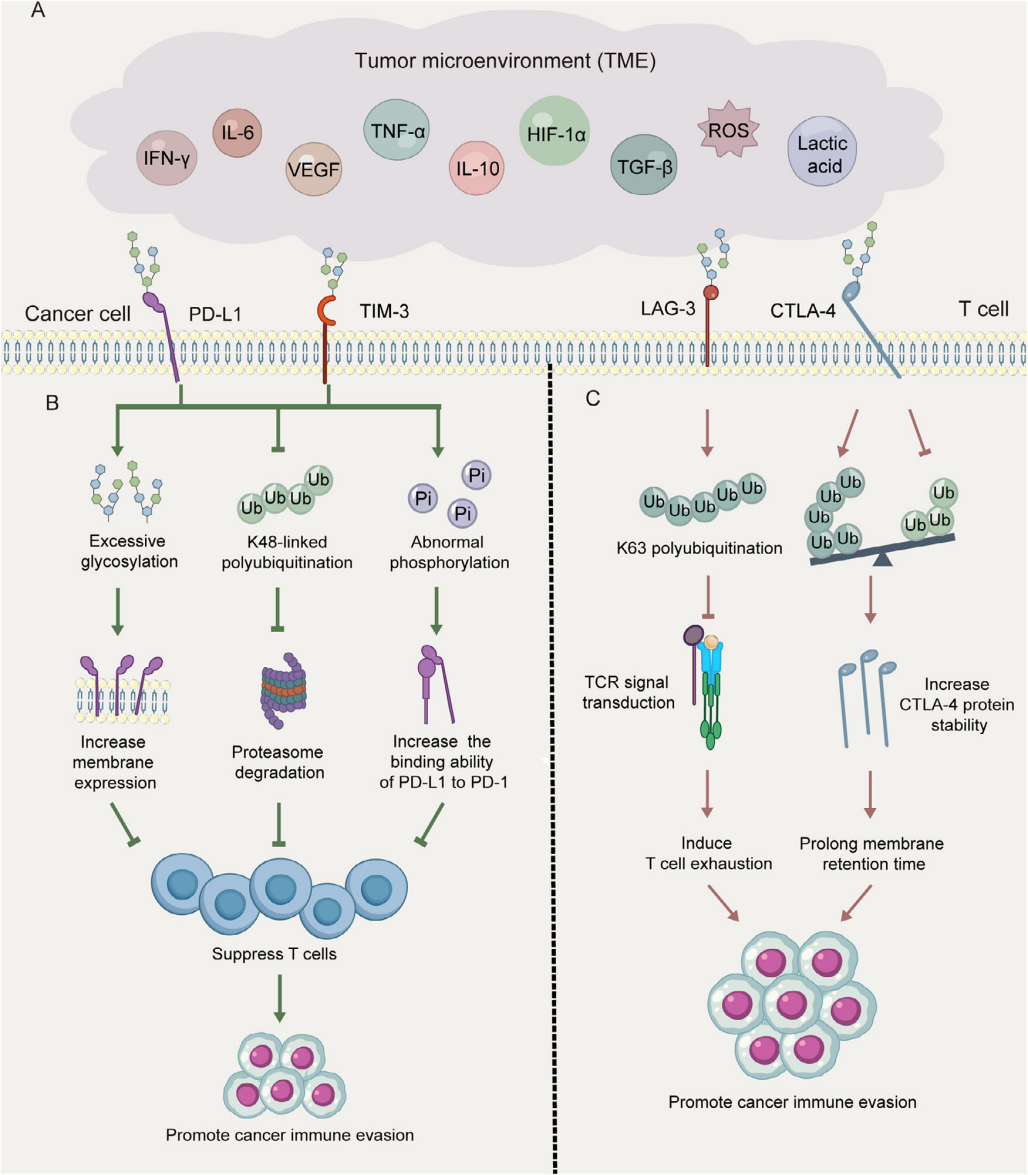
The TME affects PTMs of ICs and regulates cancer progression. (A) Cytokines and metabolites in the TME drive the upregulation and PTMs of ICs on cancer cell surfaces and T cells. (B) Abnormal PTMs of PD‐L1 and TIM‐3 on tumor cells impair the immune response. (C) Ubiquitination modifications of LAG‐3 and CTLA‐4 on T cells induce T‐cell exhaustion and promote cancer immune evasion. CTLA‐4, CTL‐associated protein‐4; HIF‐α, hypoxia‐inducible factor‐α; ICs, immune checkpoints; IFN‐γ, type‐I interferons‐γ; LAG‐3, lymphocyte activation gene‐3; PD‐L1, programmed death ligand‐1; PTMs, posttranslational modifications; ROS, reactive oxygen species; TGF‐β, transforming growth factor‐β; TIM‐3, mucin‐domain containing‐3; TME, tumor microenvironment; TNF‐α, tumor necrosis factor‐α; VEGF, vascular endothelial growth factor.

ICs, as core mediators of immune homeostasis, are vital for preventing the immune system overactivation and establishing peripheral tolerance. Nevertheless, in the setting of cancer, these checkpoints are frequently hijacked by tumors, including programmed cell death‐1 (PD‐1), programmed death ligand‐1 (PD‐L1), T cell immunoglobulin and mucin‐domain containing‐3 (TIM‐3), T cell immunoreceptor with Ig and ITIM domains (TIGIT), and so on, thereby dampening anti‐cancer immune responses and facilitating immune evasion [218]. PTMs provide an extra tier of regulation by impacting checkpoint receptor dynamics, subcellular localization, receptor‐ligand affinity, membrane retention, and downstream suppression signals, allowing checkpoints to promptly respond to extrinsic signals without perturbing gene expression (Figure 6) [219].

Following binding to PD‐L present on cancer cells or APCs, PD‐1 transmits suppressive signals that weaken CTLs activation and facilitate tumor immune evasion in cancer [220]. Phosphorylation occurs at PD‐1 Tyr248 to inhibit the TCR signaling pathway, thereby suppressing T‐cell proliferation and activation [221]. Glycosylation at PD‑1 residues N49, N58, N74 and PD‑L1 residues N192, N200, N219 can enhance their stability and interaction, thereby strengthening the immunosuppressive signal and ultimately promoting immune evasion [222]. However, ubiquitination of Lys210 and Lys233 promotes degradation of PD‐1 by the proteasome, and weakens immunosuppression and enhances anti‐tumor immunity [223]. In addition, acetylation of Lys263 prevents PD‐L1 from translocating from the plasma membrane to nucleus, and the upregulation of nuclear PD‐L1 helps cancer cells evade immune surveillance [224].

Tim‐3 is an IC that downregulates TCR signals and cytokine production, consequently promoting immune tolerance to cancer [225]. Phosphorylation at the Y256 site of Tim‐3 recruits SH2 domain kinases, thereby inhibiting TCR signaling and cytokine production, ultimately accelerating tumor progression [226]. TIM‐3 and PD‐1 glycosylation promotes the assembly of TIM‐3/Galectin‐9/PD‐1 complexes at immune synapse, significantly enabling cancer cells to evade immune surveillance [227]. Phosphorylation of Tyr225 and Tyr231 on the TIGIT molecule inhibits the AKT/MAPK/NF‐κB signaling pathway, thereby facilitating T cell and NK cell exhaustion and suppressing anti‐tumor immune responses [228]. Moreover, hypomethylation of the promoter region enhances TIGIT transcription in TILs [229].

In addition, the PTMs of other ICs are all involved in and mediate the pathological processes of malignant tumors. For example, phosphorylation of the Tyr201 of CTL‐associated protein‐4 (CTLA‐4) is catalyzed by Lck, inducing T cell inactivation [230]. Ubiquitination at the Lys498 site of lymphocyte activation gene‐3 (LAG‐3) promotes its immunosuppressive function [231]. Deacetylation of lysine residues on signal regulatory protein alpha (SIRPα) drives M2 macrophages and maintains cancer cell survival [232].

#### 6.1.2 Reshape of the TME by PTMs

TME is an intricate and dynamic environment comprising angiogenic vascular cells (AVCs), infiltrating immune cells (IICs), and cancer‐associated fibroblasts (CAFs), whose immunosuppressive characteristics are an important basis for tumor occurrence, invasion, and metastasis [233]. PTMs of different target proteins in the TME, as well as abnormal PTMs, significantly affect cancer occurrence and development by regulating the malignant phenotype of tumor cells, remodeling immune cell functions, and influencing intercellular signaling.

Various PTMs widely present within AVCs can dynamically regulate the process of tumor angiogenesis, thereby participating in the development of cancer. Vascular endothelial growth factor receptor 2 (VEGFR2) possesses potent tyrosine kinase activity. Upon binding to VEGF, it mediates various downstream AKT/MAPK signaling molecules phosphorylation, thereby promoting angiogenesis and cancer cell migration within TME [234]. VEGFR2 ubiquitination is a prerequisite for its internalization. It enhances angiogenesis and drives cancer progression via modulating its membrane expression levels [235]. PTMs occurring in IICs affect their anti‐tumor immune effects by modulating the behavior of various immune cells in TME. Abnormal SUMOylation of IRF4 can cause dysfunction of Treg cells, disrupting immune balance and accelerating tumor occurrence and development [236]. The methyltransferase SMYD3 promotes Foxp3 expression, thereby promote tumor immune evasion by directly affecting Treg differentiation [237]. c‐MAF, as a Th2‐specific factor, undergoes phosphorylation at Tyr21 and abnormal SUMOylation, which enhances anti‐tumor immunity by promoting IL‐4 production [238]. CAFs play an active function in cancer cells growth and invasion by creating a unique TME. The lactate secreted by CAFs in the TME increases, promoting lactylation of H3K18 in gastric cancer cells, thereby inhibiting antigen presentation efficiency [239]. Downregulation of N‐methyltransferase in CAFs results in diminished levels of H3K27me3 and H3K4me3. This depletion amplifies the oncogenic support capabilities of CAFs, thereby promoting the progression of ovarian cancer [240].

#### 6.1.3 Abnormal PTMs and Cancer

Aberrant translation modification interferes with protein function and signal transduction, widely contributing to tumor onset and progression, immune evasion, and therapeutic refractoriness, and is a significant molecular mechanism of cancer development.

In multiple tumors, the phosphorylation level at Tyr105 of pyruvate kinase M2 (PKM2) is greatly increased, mediating the shift of tumor cell metabolism toward aerobic glycolysis. AKT promotes breast cancer (BC) metastasis induced by TGF‐β by regulating RNF12 phosphorylation and increasing its stability. In addition, phosphorylation at Ser37 of PKM2 is a notable character of invasive BC [241]. Aberrant ubiquitination may cause harmful consequences, such as misregulation of signaling pathways, faulty assembly of protein complexes, aggregation of misfolded proteins, and improper protein localization, ultimately inducing cancer. E3 contributes to tumor initiation and progression by mediating the stabilization of oncogenes and tumor suppressor proteins [242]. Lysine succinylation imbalances may drive tumorigenesis [243]. S100A10 exhibits high levels of succinylation at the K47 site, a process catalyzed by carnitine palmitoyltransferase 1A (CPT1A), which amplifies its expression in gastric cancer and modulate the proteolysis of S100A10, subsequently fostering the aggression and metastasization of gastric cancer cells. Evidence indicates that proteins in BC are highly succinylated, particularly in histone H2A. Meanwhile, the modulation of this complex is linked to the DDR in BC [244]. Abnormal histone modifications are the core epigenetic mechanisms driving tumorigenesis, remodeling the TME, and inducing immune evasion. With the advancement of high‐throughput multi‐omics technologies, personalized epigenomics is devoted to accurately mapping patient‐specific histone dynamic modification profiles [245].

### 6.2 Autoimmune Diseases

Autoimmune diseases are a highly diverse class of disorders distinguished by immune dysfunction, largely resulting from abnormal reactions by B cells and T cells to normal host tissues. PTMs of immune‐related proteins are involved in the tolerance mechanisms of the immune system, thereby affecting the progression and pathogenesis of autoimmune diseases, including systemic lupus erythematosus (SLE), multiple sclerosis (MS), type 1 diabetes mellitus (T1D), and so on [10].

SLE represents a multi‑organ autoimmune disease whose hallmark is the production of autoantibodies against a variety of nuclear antigens. Under tolerogenic states, such as TCR binding in the absence of costimulation in patients with SLE, E3 molecules are recruited through calcium‐dependent pathways and inhibit T‐cell activation by disrupting TCR signaling [246]. During the onset of SLE, abnormal N‐glycans disrupt the normal spatial arrangement and geometric orientation of MHC II molecules in immunological synapse, significantly reducing the capability of antigen peptide presentation and the transmission of T‐cell activation signals, thereby affecting disease progression [247]. MS is an inflammatory demyelinating disorder driven by autoimmunity to myelin proteins and progressive axonal damage. The levels of myelin basic protein (MBP) and glial fibrillary acidic protein (GFAP) citrullination in MS lesion areas are significantly higher than in normal white matter [248]. It is observed that in MS animal paradigms, the overexpression of the oncogene Bcl2 is primarily Arrb1‐dependent acetylation of histone H4 at the Bcl2 promoter [249]. T1D is a chronic T‐cell‐mediated autoimmune disease, in which immune cells target endogenous pancreatic cells. Citrullination of the glutamic acid decarboxylase 65 (GAD65) peptide that can be recognized in patients with T1D can enhance its binding to diabetes‐susceptible HLA alleles [250]. Histone acetylation on the Foxp3 promoter promotes gene transcription, while inhibitory modifications, such as H3K27me3, suppress Foxp3 expression. Dysregulation of these modifications may impair the inhibitory capability of Tregs in patients with T1D [251].

As mentioned above, rheumatoid arthritis (RA), systemic sclerosis (SSc), and primary biliary cirrhosis (PBC) are also finely regulated by PTMs. The loss of galactose residues on IgG of individuals with RA is an extensively documented phenomenon among N‐glycan alterations in autoimmune conditions and is commonly employed as a prognostic marker to monitor disease progression [252]. The acetylation levels in fibroblasts of SSc patients are decreased, inhibiting the expression of connective tissue growth factor (CTGF) and promoting the fibrosis process [253]. Additionally, abnormal SENP6 and SENP2 influence TLR‑related inflammation and antiviral defense in PBC by promoting deSUMOylation of IKKγ and IRF3 [253].

### 6.3 Infection

Pathogenic microorganisms, including bacteria, viruses, and parasites, can induce infectious diseases. The host defense against infection involves a complex interplay of various cell types to combat invading pathogens. To achieve precise temporal regulation of these processes, cells typically rely on PTMs, including phosphorylation, SUMOylation, glycosylation, etc., which can rapidly and reversibly regulate the structure, function, and/or binding interactions of proteins [254]. Correspondingly, certain pathogens can also hijack or manipulate PTMs of host proteins to evade immune defenses.

The phosphorylation of viral and cellular proteins has a significant influence on viral infectivity, replication, and host cellular toxicity. Following viral infection, the MAPK and Jak/STAT cascades exploit phosphorylation events triggered by viral proteins within the cell. For example, after being infected with West Nile virus (WNV), microglial cells exhibit an increasing trend in the phosphorylation levels of intracellular p38 MAPK and JNK [255]. Epstein–Barr virus (EBV) latent membrane protein 1 (LMP1) can activate the PI3K/Akt pathway by inducing the phosphorylation of Akt, and this signaling pathway regulates the actin cytoskeleton remodeling in EBV‐infected cells [256]. *Staphylococcus aureus*, a Gram‐positive pathogen, secretes tyrosine phosphatase A to dephosphorylate signaling molecules. This attenuates the SUMOylation of NF‑κB and IRF3, resulting in reduced pro‑inflammatory cytokine synthesis and macrophage activity, and inhibits inflammasome recruitment to sustain persistent infection [257]. Upon entering host cells, virus exploits glycosylation pathways to reproduce and avoid the immune system. N‐glycosylation at Asn297 in IgG molecules exhibits pronounced alterations following *Mycobacterium tuberculosis* infection [258]. Remarkably, the lysine methyltransferase SET domain bifurcated protein 2 (Setdb2) is upregulated upon viral infection, subsequently binding to and inducing repressive H3K9me3 marks on the Cxcl1 gene promoter, ultimately causing both a reduction in neutrophil infiltration and a decline in host resistance to secondary bacterial infections [259]. Spike glycoprotein ubiquitination in SARS‐CoV‐2 at Lys310, Lys986, and Lys1028 sites can significantly enhance the virus infection of lung epithelial Calu3 cells, while mutating these sites markedly reduces its infectivity [260].

## 7 Targeting PTMs for Immunotherapy

PTMs possess core features including dynamic reversibility, disease specificity, and mechanistic plasticity, and are widely located at key nodes of signaling pathways, capable of simultaneously influencing multiple immune processes, thus being targeted by SMIs [12], biologics [13], or other technological interventions. Targeting PTMs for immunotherapy offers promising new avenues and is gradually recognized as a major advance against cancer, autoimmune diseases, and infectious diseases. The main strategy involves specifically regulating enzymes associated with PTMs or directly modulating the functions of PTM‐modified immune‐related proteins, thereby correcting abnormal immune regulatory networks, restoring immune homeostasis, and ultimately achieving therapeutic effects against immune‐related diseases [216].

### 7.1 Small Molecule Inhibitors

SMIs, a core strategy for targeting PTMs in immunotherapy, feature low molecular weight, strong tissue penetration, oral availability, and mature clinical translation. SMIs, including immune checkpoint inhibitors (ICIs), HDAC inhibitors, and others, provide an efficient treatment pathway for immune‐related diseases, while also laying the foundation for overcoming the limitations of traditional immunotherapy [261]. We summarized clinical trials associated with SMIs in Table 1.

**TABLE 1:** Clinical trials associated with SMIs targeting PTMs for immune‐related diseases: molecular targets, mechanisms of action, indications, and trials status.

NCT	Drug	Target	PTM type	Mechanism of action	Indications	Phase
NCT02281552	Tofacitinib	JAK	Phosphorylation	Inhibiting JAK1/3, thereby blocking the phosphorylation activation and signal transduction of downstream STAT	RA	III
NCT04446650	Fedratinib	JAK	Phosphorylation	Inhibiting JAK2 and blocking erythropoietin and other myeloproliferative signaling	MF	I/ II
NCT04186871	Branebrutinib	BTK	Phosphorylation	Inhibiting the BTK pathway to block B cells from producing autoantibodies	RA, SLE	II
NCT03233230	Fenebrutinib	BTK	Phosphorylation	Inhibiting BTK phosphorylation activation, blocking the inflammatory signal transduction of downstream B cells	RA, MS	II
NCT05856448	GLPG3667	TYK2	Phosphorylation	Inhibiting TYK2 and blocking the phosphorylation‐mediated activation of STAT proteins in the IFN‐I signaling pathway	SLE	II
NCT05662397	HST‐1011	Cbl‐b	Ubiquitination	Inhibiting Cbl‐b to prevent the ubiquitination and subsequent degradation of key activating proteins in immune cells	Solid tumor	I/II
NCT05107674	NX‐1607	Cbl‐b	Ubiquitination	Inhibiting Cbl‐b, blocking the ubiquitin‐mediated degradation of immune signaling proteins in T cells and NK cells	Advanced malignancies	I
NCT03648372	TAK‐981	SAE	SUMOylation	Inhibiting SAE to prevent protein SUMOylation and thereby activate the IFN‐I signaling pathway	NSCLC, cervical cancer	I/ II
NCT01897571	Tazemetostat	EZH2	Methylation	Inhibiting EZH2 to relieving transcriptional repression of tumor suppressor genes	B‐cell lymphomas	I
NCT00128661	GSK‐J4	JMJD3	Methylation	Inhibiting JMJD3 demethylases to increase H3K27me3 levels, thereby inducing apoptosis in cervical cancer	Cervical cancer	III
NCT05584111	STC‐15	METTL3	Methylation	Inhibiting METTL3 to block m6A methylation modification of RNA, thereby reshaping TME	Advanced cancer	I
NCT00127127	Vorinostat	HDAC	Acetylation	Inhibiting HDAC to increase the acetylation levels of histones, thereby reactivating the transcription of tumor suppressor genes	Solid tumors	I
NCT02635061	ACY‐241	HDAC	Acetylation	Inhibiting HDAC6 to enhance the acetylation levels of tubulin and other proteins	NSCLC	I
NCT00792831	ITF2357	HDAC	Acetylation	Inhibiting HDAC thus activates the apoptotic signaling pathway	CLL	II
NCT03838926	Trichostatin A	HDAC	Acetylation	Inhibiting HDAC to reshape gene expression and induce apoptosis in HM cells	HM	I
NCT04068597	Inobrodib	p300	Acetylation	Inhibiting p300 to downregulate the expression of oncogenes and induce apoptosis in HM cells	HM	I/II
NCT03616470	Uproleselan	E‐selectin	Glycosylation	Inhibiting E‐selectin and block its adhesion to glycosylated ligands of AML cells	AML	III
NCT02952989	SGN‐2FF	FUTs	Glycosylation	Inhibiting the fucosylation modification of proteins and block oncogenic growth pathways	NSCLC	I

Abbreviations: AML, acute myeloid leukemia; BTK, Bruton's tyrosine kinase; Cbl‐b, Casitas B‐lineage lymphoma proto‐oncogene b; CLL, chronic lymphocytic leukemia; EZH2, enhancer of Zeste homolog 2; FUTs, fucosyltransferases; HDAC, histone deacetylase; HM, hematologic malignancy; JAK, Janus kinase; METTL3, methyltransferase‐like 3; MF, myelofibrosis; MS, multiple sclerosis; NSCLC, non‐small cell lung cancer; RA, rheumatoid arthritis; SAE, SUMO‐activating enzyme; SMIs, small molecule inhibitors; STAT, signal transducer and activator of transcription; SLE, systemic lupus erythematosus; TYK2, tyrosine kinase 2; TME, tumor microenvironment.

*Source*: ClinicalTrials.gov.

#### 7.1.1 Cancer Immune Checkpoint Inhibitors

ICIs are attracting growing attention in cancer research and hold promising potential for application in the field of immunotherapy. ICs are designed to prevent tissue injury and autoimmunity by moderating immune responses, but cancer cells frequently exploit them to evade eradication. The identification of ICs has been instrumental in advancing the development of cancer immunotherapies [262]. Based on their targets, ICIs include PD‐1 inhibitors, PD‐L1 inhibitors, CTLA‐4 inhibitors, and others. Exploratory research into novel ICs such as TIM‐3, VISTA, B7‐H3, BTLA, and TIGIT is continuing to advance.

Some clinically used ICIs achieve immunotherapeutic effects by affecting PD‐L and PD‐L1 expression. For example, ES‑072, an EGFR inhibitor, strongly triggers PD‐L1 ubiquitination modification and subsequent degradation [263]. The specific OTUB2 inhibitor OTUB2‐IN‐1 inhibit its deubiquitinating activity without interfering with the OTUB2‑PD‑L1 complex formation, thereby driving PD‐L1 downregulation in cancer cells and restraining malignant progression [264]. The OTUD3 inhibitor rupatadine competes for binding with OTUD3, thereby reducing deubiquitination of PD‑L1 mediated by OTUD3 and MYL12A [265]. Angustmycin A, as a guanosylate synthetase (GMPS) inhibitor, markedly suppresses PD‐L1 levels by preventing GMPS‑driven PD‐L1 glycosylation, thereby halting hepatocellular carcinoma malignancy and enhancing the sensitivity to anti‐CTLA‐4 antibodies [266]. Bezafib has demonstrated new anticancer potential in clinical applications for hyperlipidemia treatment. By increasing the expression of CPT1A, it exerts a cooperative effect with anti‐CTLA‐4 antibodies, ultimately inhibiting cancer growth [267]. Oligosaccharide transferase inhibitors NGI‐1 augment the body response to immunotherapy by downregulating the PD‐L1 N‐glycosylation [268]. Notably, the FDA authorized the monoclonal antibody relatlimab, which targets LAG‐3, together with nivolumab for metastatic melanoma therapy in 2022 [269].

#### 7.1.2 HDAC Inhibitors

In terms of acetylation modifications, HDAC inhibitors were the first epigenetic drugs to be successfully used in clinical practice. Vorinostat, an FDA‑approved therapy for refractory cutaneous T‑cell lymphomas, acts as an antitumor agent by re‑establishing histone acetylation levels and reprogramming gene expression. It also demonstrates favorable safety profiles and synergistic antitumor activity when used in combination [270]. Romedicin, a further HDAC inhibitor sanctioned by the FDA, is indicated for the management of cutaneous and peripheral T‑cell lymphomas. Romedicin has been employed in various Phase II and early‐stage clinical trial settings to perform ongoing monitoring of acetylation levels in patients peripheral blood mononuclear cells [271]. Furthermore, chidamide and CM‐1758 promote PD‐L1 histone acetylation. Their synergistic use with PD‐1/PD‐L1 inhibitors results in effective tumor regression [272].

#### 7.1.3 The Other Inhibitors

As the first FDA‐approved UPS inhibitor, bortezomib has become an important drug for the treatment of multiple myeloma [273]. ONX‐0914, an immunoproteasome inhibitor, has shown therapeutic potential for various autoimmune diseases [274]. Another immunoproteasome inhibitor, KZR‐616, has received approval for Phase Ib/II clinical studies involving SLE patients [275]. TAK‐981, a clinical candidate molecule, is an inhibitor of the SUMO‐activating enzyme. It acts as an inhibitory active substance by forming a SUMO‐TAK‐981 adduct at the catalytic site of enzyme. It has shown potential antitumor activity in preclinical tumor models [276]. P5091, as a USP7 inhibitor, amplifies macrophage antigen‐presenting capacity and promotes T‐cell activation, making it suitable for immunotherapy against viral infections such as hepatitis B and COVID‐19 [277]. Ruxolitinib, by blocking the JAK/STAT signaling pathway, boosts macrophage and NK cell activity, thereby promoting the clearance of intracellular pathogens. It can serve as an adjunctive therapy for *Mycobacterium tuberculosis* and Brucella infections [278].

### 7.2 Biologics

Compared with SMIs, biologics targeting PTMs have the advantages of high specificity, strong targeting, low side effects, and good biocompatibility, mainly including monoclonal antibodies(mAb), antibody‐drug conjugates (ADC), fusion proteins, chimeric antigen receptor T‐cell (CAR‐T) therapy, and vaccines, among which mAbs are the type of biologic agents approved by the FDA [279]. We summarized clinical trials associated with biologics in Table 2.

**TABLE 2:** Clinical trials associated with biologics targeting PTMs for immune‐related diseases: molecular targets, mechanisms of action, indications, and trial status.

NCT	Drug	Target	PTM type	Mechanism of action	Indications	Phase
NCT05424380	GSK3745417	SAE	SUMOylation	Inhibit SUMO‐specific enzymes to block the SUMOylation of key proteins, thereby disrupting survival signals in AML cells	AML	I
NCT02472145	Decitabine	DNMT1	Methylation	Inhibiting DNA methyltransferases to reduce the DNA methylation levels in AML cells and inducing cell apoptosis	AML	II/III
NCT00413478	Azacitidine	DNMTs	Methylation	Reducing the DNA methylation levels of CLL cells to reactivate silenced tumor suppressor genes	CLL	II
NCT00256334	Resveratrol	SIRT1	Acetylation	Activating SIRT1 to reduce the acetylation levels of p53 or NF‐κB, thereby inhibiting the proliferation of colon cancer cells	Colon cancer	I
NCT02281409	Mogamulizumab	CCR4	Glycosylation	Utilizing its Fc region to defucosylate, significantly enhancing the ADCC effect, thereby activating anti‐tumor immunity	Solid tumors	I/ II
NCT00576758	Obinutuzumab	CD20	Glycosylation	Utilizing its Fc region after afucosylation to achieve high affinity with FcγRIIIa, significantly enhances the ADCC effect	Non‐Hodgkin's lymphoma	II
NCT03381170	Ublituximab	CD20	Glycosylation	Utilizing its Fc region, after defucosylation, significantly enhances ADCC effects, to efficiently deplete pathogenic B cells	MS	II

Abbreviations: SAE, SUMO‐activating enzyme; AML, Acute Myeloid Leukemia; DNMT1, DNA methyltransferase 1; CLL, Chronic lymphocytic leukemia; SIRT1, Silent mating type information regulation 2 homolog 1; CCR4, C‐C chemokine receptor type 4; ADCC, Antibody‐dependent cellular cytotoxicity.

*Source*: ClinicalTrials.gov.

#### 7.2.1 Monoclonal Antibodies

The potency of mAb in cancer therapy relies on the tumor antigen profile and the unique signaling pathway targeted by the specific antibody. Trastuzumab and pertuzumab are two classic anti‐HER2 mAb. In particular, pertuzumab particularly inhibits both heterodimerization and homodimerization between HER2 and EGFR. The inhibition disrupts downstream signal transduction, thereby suppressing the abnormal cell proliferation driven by receptor activation [280]. Lpilimumab, the first anti‐CTLA‐4 mAb to enter clinical trials, was approved in 2011 for malignant melanoma therapy [281]. The STM108 antibody specifically recognizes certain glycosylation sites on PD‐L1 protein and effectively inhibits the PD‑L1/PD‑1 interaction by binding to N35, N192, and N200 [282]. Belimumab is a humanized monoclonal antibody specifically targeting B‐lymphocyte stimulator, and it received FDA approval for SLE therapy. It regulates immune balance by inhibiting B‐cell activation, differentiation, and autoantibody production [283]. ARV‐771 can combine a chimeric structure that induces target protein degradation with anti‐PD‐L1 antibody, thereby inducing PD‐L1 ubiquitination. This overcomes resistance to PD‐L1 inhibitors and shows good antitumor activity in triple‐negative BC [284].

#### 7.2.2 The Other Biologics

EMMAE was conjugated with a high‐affinity anti‐human CD6 mAb to prepare a CD6‐targeted ADC, which can effectively and selectively kill T‐cell lymphoma cells in vitro [285]. CD45‐ADC, which is directed at hematopoietic cells, can precisely deplete both host hematopoietic stem cells and immune cells, making it possible to achieve donor hematopoietic stem cell engraftment [286]. Fc fusion activates immunomodulatory functions via the Fc fragment, thereby relieving the inflammatory symptoms of SLE [287]. Preclinical research have indicated that CD19‐directed CAR‐T cell therapy substantially attenuates inflammation of the synovial membrane and erosive bone loss in RA [288]. The ASPIRE nanovaccine employs DCs infected with a reconstituted adenovirus to target the anchor MHC I, which amplify antigen presentation to lymphatic tissues and elicit a broad‐spectrum antitumor T‐cell response.

### 7.3 Others

In addition to SIMs and biologics, immunotherapy targeting PTMs also encompasses directions, including molecular glue degraders, nucleic acid drugs, gene editing technologies, and peptide inhibitors. These directions have become research hotspots in recent years due to their unique functional advantages.

Bardoxolone‐methyl (CDDO‐Me) is a triterpenoid molecular glue that induces the autophagic lysosomal degradation of EGFR by simultaneously attaching to EGFR and KEAP1, regulating the ubiquitination and phosphorylation modifications of EGFR. It shows good antitumor effects in a triple‐negative BC orthotopic transplantation model [289]. siRNA‐STAT3 silences *STAT3* gene expression and inhibits STAT3 phosphorylation activation, thereby reducing the secretion of immunosuppressive molecules. It boosts the cytotoxic activity of T and NK cells and has been applied in preclinical models of non‑small cell lung cancer and colorectal cancer. Combined with PD‐L1 inhibitors, it can significantly improve antitumor effects [290]. Approved by the FDA for the clinical treatment of cutaneous T‑cell lymphoma, romidepsin is a cyclic peptide that acts as a broad‑spectrum and potent inhibitor of histone deacetylase [291]. In addition, the study demonstrated a TME‐activated nanoassembly system, which, by co‐assembling PD‐L1 with CTLA‐4 antagonistic aptamers onto a glucose transporter 1 (GLUT1) inhibitor, was confirmed to significantly reduce the N‐linked glycosylation level of PD‐L1 [292].

## 8 Challenges, Limitations, and Future Perspective

Although researchers have comprehensively delineated the types of PTMs in the immune system and their regulatory mechanisms, numerous fundamental questions regarding how PTMs regulate immune responses remain unanswered [293]. In particular, the crosstalk, both synergistic and antagonistic, between distinct PTM types, the PTM‐encoded functional heterogeneity of immune cells, and the spatiotemporal rewiring of these modifications across different immune stages and pathological contexts are key unresolved issues.

The highly dynamic, transient, and low‐abundance nature of PTMs imposes fundamental limits on conventional mass‐spectrometry‐based proteomics. Capturing fleeting modification events and resolving PTMs heterogeneity across immune cell subpopulations at single‐cell resolution remain formidable analytical challenges [218]. These technical constraints, compounded by the intrinsic complexity of PTMs regulatory logic, severely hinder mechanistic dissection. Immune responses are orchestrated through elaborate crosstalk among diverse modification types. For instance, in the TME, key immune molecules can be simultaneously modified by phosphorylation, ubiquitination, glycosylation, and lactylation. These marks can antagonize or synergize, collectively dictating the activation, differentiation, and exhaustion states of immune cells [294]. Recent evidence further demonstrates that heterogeneous tumor‐derived metabolites can directly rewire the epigenetic landscape of neighboring immune cells through unconventional PTMs such as lactylation. This deep metabolic–modification coupling adds an additional layer of complexity to an already intricate regulatory network [295].

A deep mechanistic understanding of PTM‐mediated immune regulation unveils compelling therapeutic targets for cancer and other diseases, yet clinical translation remains profoundly challenging. The enzymes that catalyze PTMs are broadly engaged in normal physiology. Consequently, conventional systemic SMIs frequently lack the requisite selectivity and carry a substantial risk of severe systemic toxicity [296]. In parallel, achieving efficient, targeted delivery of antibodies or nucleic acid‐based therapeutics directed against specific PTMs states across complex in vivo barriers represents a critical unmet need. Despite these formidable obstacles, drug development aimed at protein PTMs holds immense promise. Poor selectivity has long been a primary driver of adverse effects, prompting the development of more selective HDAC inhibitors and covalent inhibitors capable of engaging defined protein conformations, thereby sharpening target discrimination and curbing off‐target liabilities [297]. The emergence of targeted protein degradation (TPD) has fundamentally altered the therapeutic landscape: by enabling the degradation of proteins long considered undruggable, it has rapidly become a focal point of PTM‐related research [298]. Proteolysis‐targeting chimeras (PROTACs), bifunctional hybrid molecules, recruit a protein of interest to endogenous E3 ubiquitin ligases, triggering its ubiquitination and subsequent proteasomal destruction [299]. In parallel, nanodelivery systems can amplify therapeutic efficacy by optimizing pharmacokinetics, prolonging tunable drug release, enabling tissue‐specific targeting, and facilitating intracellular cargo discharge [300]. Integrating such delivery platforms with PTMs modulators represents another important frontier.

In the future, with the development of multi‐omics integrative analysis, single‐cell and spatial proteomics, structural biology, and artificial intelligence‐assisted drug discovery, the regulatory network of PTMs in immunity is expected to be further elucidated, thereby providing new theoretical foundations and therapeutic targets for precise immune interventions.

## 9 Conclusion

PTMs, as reversible and dynamic processes, greatly broaden the spectrum of protein functions under physiologic and pathologic circumstances by modifying protein structure, activity, electrical charge, thermal stability, and their interplay with intracellular and extracellular DNA, RNA, and other proteins, as well as intricate crosstalk. The immune system, composed of immune organs/tissues, immune cells, and immune molecules, can be divided into innate immunity and adaptive immunity (the latter including T‐cell‐mediated cellular immunity and B‐cell‐mediated humoral immunity), coordinately exerting immune defense and immune surveillance, while maintaining immune homeostasis. PTMs accurately regulate the biological activity of immune‐related proteins and widely participate in the immunological response, including antigen recognition, immune signal transduction, antigen elimination, antibody production and class switching, immune contraction, and the establishment of immune memory.

With the rapid advancement of research on PTMs, increasing evidence shows that abnormal PTMs are involved in the occurrence and development of various diseases, including cancer, autoimmune diseases, and infectious diseases. Elucidating the mechanisms that govern PTMs not only deepens our understanding of disease pathogenesis but also provides promising therapeutic targets and novel strategies for clinical management. Targeting PTMs has become an important strategy for treating immune‐related diseases, leading to the development of various SMIs, biologics, and other targeted interventions for clinical practice.

Although targeting PTMs shows great potential in the treatment of immune‐related diseases, it still faces many challenges. The interactive PTMs regulatory network is highly complex and dynamic, making precise control difficult with single‐target interventions. Moreover, the highly conserved structure of PTM regulatory enzyme families makes it challenging to design selective small‐molecule drugs. The robust intervention in PTMs may also trigger uncontrolled normal immune responses, resulting in autoimmune‐like adverse effects. This may stem from the absence of suitable biomarkers, which precludes real‐time monitoring and compromises the ability to sustain therapeutic efficacy.

We believe that with the rapid development of new technologies and algorithms, the repertoire of PTMs may still increase, which will help to elucidate the code of life. Furthermore, clarifying the intrinsic connections of proteomics, metabolomics, and epigenomics with cancer, autoimmune diseases, and infectious diseases is a prerequisite for making breakthroughs. While focusing on key disease‐driving factors and integrating AI‐assisted network target screening, TPD technology, and nanodelivery systems, will promote the rapid evolution of this field. Taken together, despite the considerable challenges ahead, the future of PTM‐targeted therapies for immune‐related diseases is decidedly bright.

## Author Contributions

Yuanyan Liu and Yuxing Yao conceived the study and developed the framework. Jinxiu Qian, Qiqiong Liu, Wanting Ye, Ying Xing, Xiuyun Bai, Rongjun Deng, and Jue Yang conducted the literature search and data collection. Cheng Lyu, Cheng Xiao, Yuanyan Liu, and Yuxing Yao drafted the manuscript, and all authors critically reviewed and revised it. All authors read and approved the final manuscript.

## Ethics Statement

The authors have nothing to report.

## Conflicts of Interest

The authors declare no conflicts of interest.

## Data Availability

The authors have nothing to report.
